# Ready for Repair? Gene Editing Enters the Clinic for the Treatment of Human Disease

**DOI:** 10.1016/j.omtm.2020.06.022

**Published:** 2020-07-03

**Authors:** Martijn P.T. Ernst, Mike Broeders, Pablo Herrero-Hernandez, Esmee Oussoren, Ans T. van der Ploeg, W.W.M. Pim Pijnappel

**Affiliations:** 1 Department of Pediatrics, Erasmus University Medical Center, Rotterdam, the Netherlands; 2 Department of Clinical Genetics, Erasmus University Medical Center, Rotterdam, the Netherlands; 3 Center for Lysosomal and Metabolic Diseases, Erasmus University Medical Center, 3015 GE Rotterdam, the Netherlands

## Abstract

We present an overview of clinical trials involving gene editing using clustered interspaced short palindromic repeats (CRISPR)-CRISPR-associated protein 9 (Cas9), transcription activator-like effector nucleases (TALENs), or zinc finger nucleases (ZFNs) and discuss the underlying mechanisms. In cancer immunotherapy, gene editing is applied *ex vivo* in T cells, transgenic T cell receptor (tTCR)-T cells, or chimeric antigen receptor (CAR)-T cells to improve adoptive cell therapy for multiple cancer types. This involves knockouts of immune checkpoint regulators such as PD-1, components of the endogenous TCR and histocompatibility leukocyte antigen (HLA) complex to generate universal allogeneic CAR-T cells, and CD7 to prevent self-destruction in adoptive cell therapy. In cervix carcinoma caused by human papillomavirus (HPV), E6 and E7 genes are disrupted using topically applied gene editing machinery. In HIV infection, the CCR5 co-receptor is disrupted *ex vivo* to generate HIV-resistant T cells, CAR-T cells, or hematopoietic stem cells. In β-thalassemia and sickle cell disease, hematopoietic stem cells are engineered *ex vivo* to induce the production of fetal hemoglobin. AAV-mediated *in vivo* gene editing is applied to exploit the liver for systemic production of therapeutic proteins in hemophilia and mucopolysaccharidoses, and in the eye to restore splicing of the CEP920 gene in Leber’s congenital amaurosis. Close consideration of safety aspects and education of stakeholders will be essential for a successful implementation of gene editing technology in the clinic.

## Graphical Abstract

**Figure:**
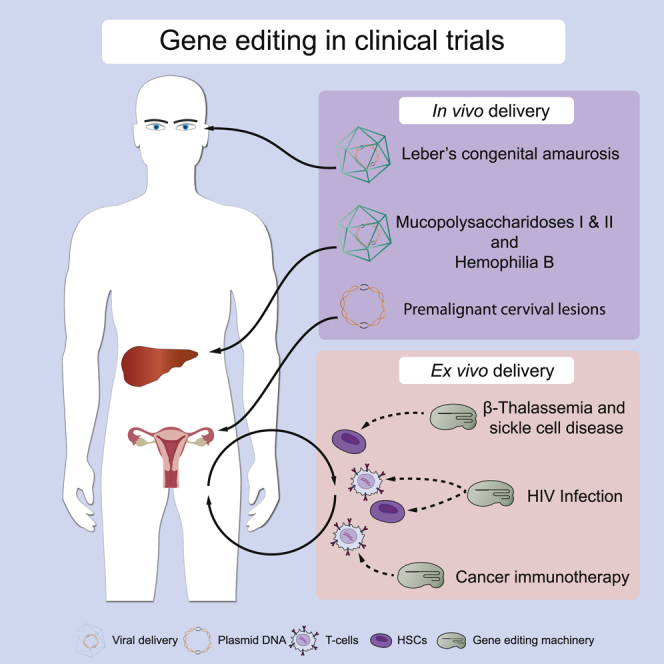

## Teaser

Much effort is dedicated to utilize gene-editing technology for the development of treatment options for human disease. This has already resulted in the first clinical trials using gene-editing tools. In this review, Pijnappel and colleagues present an overview of clinical trials involving gene editing and discuss the underlying mechanisms.

## Main Text

### Conventional Gene Therapy

Traditionally, gene therapy relies on viral-based delivery of a protein-coding gene that either semi-randomly integrates into the genome (for retroviruses and lentiviruses) or remains as extrachromosomal DNA copy (for adeno-associated virus [AAV]).^1^, ^2^, ^3^ These forms of gene therapy usually use overexpression of a protein that is missing or mutated in human disease. Lentiviral gene therapy has the advantage of being highly efficient and causing long-term efficacy. A drawback of lentiviral gene therapy is the lack of control of the location at which the virus integrates into the host genome, with the risk of insertional mutagenesis. By optimizing the lentiviral backbone and by controlling the number of viral copies, it has been demonstrated in multiple clinical trials that lentiviral gene therapy is safe provided that it is used with the proper precautions.^2^^,^^4^ AAV-mediated gene therapy does not rely on integration into the host genome but instead involves delivery of a DNA episome to the nucleus. It is therefore considered to have a lower risk of genotoxicity compared to lentiviral gene therapy. However, episomal copies of AAV DNA are lost upon cell division, resulting in loss of efficacy. This restricts AAV gene therapy to nondividing cells. In addition, pre-existing immunity to AAV capsid proteins occurs in a significant percentage of the human population and precludes eligibility for the treatment.^5^ Acquired immunity after a single AAV-mediated gene therapy treatment occurs invariably in patients and precludes eligibility for a second treatment. In both forms of gene therapy, cDNA overexpression can only be used when dosage effects of the transgene product do not apply. Although the desired average number of gene copies can be approached via the viral titer, it is not possible to precisely control this using viral-based overexpression.

### Basics of Gene Editing

Developments in recent years have enabled the seamless engineering of the human genome using a variety of tools collectively termed gene editing. Precision gene editing strategies allow alteration of the genome of cells at specific loci to generate targeted genomic changes, which are being exploited for multiple applications in medicine. We first introduce the basics of gene editing and then summarize the major challenges for their clinical implementation. Gene editing tools that are currently under investigation in clinical trials include zinc finger nucleases (ZFNs), transcription activator-like effector nucleases (TALENs), and clustered interspaced short palindromic repeats (CRISPR) in combination with CRISPR-associated protein (Cas). For a detailed comparison between these tools, we refer to previously published reviews.^6^^,^^7^ In short, target site recognition occurs by sequence-specific DNA-binding proteins (in the case of ZFNs and TALENs) or by a short stretch of RNA termed single guide RNA (sgRNA; in the case of CRISPR-Cas). Current clinical applications of gene editing rely on the introduction of double-strand DNA breaks (DSBs), mediated by Fok-1 (in the case of ZFNs or TALENs) or by Cas nucleases (in the case of CRISPR-Cas) and the introduction of desired genomic alterations through the cell’s endogenous DNA repair mechanisms. Two major DNA repair pathways are being exploited to conduct targeted genomic changes in clinical trials: (1) gene editing through homology-directed repair (HDR) used to replace a pathogenic variant or insert foreign DNA elements to restore the wild-type (WT) expression of a missing (or truncated) gene; and (2) non-homologous end joining (NHEJ) used to remove DNA elements leading to aberrant expression of genes or to gain a therapeutic function.

In contrast to traditional strategies for gene therapy, gene editing provides more versatile tools for gene therapy, for example to precisely correct point variants,^8^^,^^9^ to place an extra, healthy gene copy at a safe genomic location of choice (a safe harbor: a location in the human genome at which integration of a gene is not harmful),^10^^,^^11^ or to disrupt a gene. This would, for example, enable the restoration of endogenous expression levels following precise correction of the disease-associated variant within the natural locus, which would be especially important for gene products for which a correct dosage is required. It would also increase control of integration sites of a cDNA by choosing appropriate safe harbor locations. Such locations also should provide efficient transcription of the transgene by providing a favorable epigenetic environment consisting of euchromatin. Examples of safe harbor locations in the human genome are the albumin, *AAVS1*, and the *CCR5* loci.

### On-Target or Off-Target?

Although the technology for gene editing is rapidly evolving, there are still important challenges for its clinical implementation. First, undesired editing of genomic regions can occur as a side effect of gene editing.^7^ This can be off-target, i.e., the introduction of a DNA break outside the genomic region of choice due to the targeting of the gene editing machinery to a chromosomal location that carries sequence similarity to the region of interest. In this scenario, genes or regulatory regions other than the targeted gene can be modified, resulting in undesired downstream effects. Undesired events may include insertions, deletions, and chromosomal translocations.^12^^,^^13^ Undesired variants can also be generated on-target, i.e., unintended modification of the genomic region of interest. In this scenario, regulatory elements within the gene of interest may be unintentionally changed. This may include elements involved in promoter activity, splicing, mRNA stability, protein translation, or microRNA (miRNA) genes (that are often present in introns or untranslated regions). The CRISPR-Cas9 system is inherently more prone to off-target effects compared to ZFNs or TALENs, because target site recognition in CRISPR-Cas9 relies on RNA-DNA interaction of only short stretches, and the RNA-DNA interaction allows some mismatches. In contrast, ZFNs and TALENs depend on highly specific protein-DNA interactions that allow fewer mismatches.^14^ This has promoted much research directed toward enhancing the performance of CRISPR-Cas-based gene editing with respect to specificity and nuclease activity (see below). Methods to detect undesired events in gene editing often rely on *in silico* predictions, followed by analyses of predicted off-target events. This is not necessarily sufficient for clinical application, and unbiased analysis based on next-generation sequencing is expected to become an important tool in the future. For a more extensive discussion on off-target effects, see Broeders et al.,^7^ Kim et al.,^15^ Manghwar et al.,^16^ and Pattanayak et al.^17^

### Delivery of Gene Editing

The delivery of gene editing tools is a crucial aspect when it comes to clinical implementation. Two routes can be distinguished: *ex vivo* and *in vivo* delivery.^18^^,^^19^ In *ex vivo* delivery, autologous or allogeneic cells are modified by gene editing outside the patient, and gene-modified cells are transplanted into the patient. Any route of administration of gene editing machinery can be applied *ex vivo*, such as transfection, nucleofection, or (viral) transduction. *Ex vivo* gene editing allows quality control prior to treatment. In particular, undesired off-target and on-target events can be monitored. Note that quality control can be performed on bulk generations of cells. Rare undesired events that occur in only a few cells and that might cause cellular transformation will be difficult to detect. Alternatively, this method involves an extra complication: the engraftment of (stem) cells. For example, maintaining engraftment potential and viability of the cell of interest can be challenging. Clinically, the most advanced forms of *ex vivo* gene editing involve T cells and hematopoietic stem cells (HSCs). In *in vivo* gene editing, gene editing tools are applied directly to the organism. Vehicles for delivery include AAV, lipid nanoparticles (LNPs), gold nanoparticles (GNPs), or cell-penetrating peptides (CPPs). The delivery method in *in vivo* gene editing is crucial for its safety.^20^ When gene editing components are delivered *in vivo* via vehicles that remain present for an extended period, for example via AAV, there is a cumulative risk of undesired genotoxic events that can last for the time that the AAV remains present, which has been estimated to last for a period of 10 years or longer.^1^ In contrast, when delivered as RNA or protein, there is only short-term exposure and a reduced risk of genotoxicity.

For *in vivo* gene editing, immunity against the delivery vehicle and the gene editing components are important considerations.^21^ Both pre-existing and acquired immunity should be considered. The AAV delivery vehicle is subject to pre-existing immunity in a significant proportion of the population.^1^ In addition, preexisting immunity to Cas9 protein from several species has been reported in several studies. This may neutralize the therapy or induce adverse events.^21^, ^22^, ^23^

In summary, the safety and efficacy of gene editing technology for the treatment of human disease depend on multiple factors, including the choice of the gene editing method, being either *ex vivo* or *in vivo*, the gene editing technique, target site selection, delivery method, and target tissue.

### Gene Editing 2.0: Preclinical Developments

Technological developments are ongoing to improve gene editing tools with respect to specificity, efficiency, and versatility. These have been extensively described by us and others in recent reviews^7^^,^^24^, ^25^, ^26^ and are only briefly mentioned here.

First, variations of the original CRISPR-Cas9 method have been designed. These include the following: homology-independent targeted integration (HITI) for generating a knockin via NHEJ without involvement of HDR;^27^ microhomology-mediated end joining (MMEJ)-dependent knockin, which is based on the presence of short stretches of homology that are utilized by the MMEJ DNA repair pathway;^28^ base editing,^29^ a mismatch repair- or base excision repair-dependent pathway in which a natural cytidine or adenosine deaminase (ADA) is coupled to a catalytically dead Cas9 (dCas9) to convert cytidine to uridine (which is replicated as thymidine), or to convert adenine to inosine, which is replicated as guanine; and prime editing,^30^ in which a Cas9 nicking variant is used that introduces single stranded DNA breaks and that is coupled to reverse transcriptase to enable a wide variety of genomic changes. Second, other natural and engineered Cas9 variants have been identified and developed with distinct and/or enhanced targeting properties, including Cas12a (Cpf1), Cas12b (C2c1), FokI fused to dCas9,^31^ Cas9-HF1,^32^ eSpCs9,^33^ evoCas9,^34^ and HypaCas9.^35^ Third, Cas9 variants with distinct protospacer-adjacent motif (PAM) recognition sites have been generated, including VQR and VRER variants, xCas9, and SpCas9-NG.^36^ And fourth, sgRNAs have been modified with respect to their length, structure, and chemistry to reduce off-target properties.^37^, ^38^, ^39^ These promising developments need future work to evaluate their suitability for clinical testing.

### Scope of This Review

Whereas there have been numerous applications of gene editing in preclinical studies, information on clinical applications of gene editing is scattered in the literature. In this review, we present a comprehensive overview of current clinical trials using gene editing strategies for the treatment of human disease, and include selected preclinical examples. For more extensive overviews of preclinical studies, we refer to excellent reviews.^40^^,^^41^ In addition, in this review, we focus on gene editing in somatic cells, and we refer to other recent reviews and opinion articles for editing the germline.^42^, ^43^, ^44^ Thus far, precision gene editing has entered the clinic for the treatment of cancer immunotherapy, viral infections, and inherited hematologic, metabolic, and eye disorders (Table 1). These trials along with the underlying strategies are described in more detail below.

**Table 1:** Current Clinical Trials Involving Gene Editing

Title	Tool	Status	Country	Delivery	ID	Ref.
**Cancer Immunotherapy**						

PD-1 knockout engineered T cells for advanced esophageal cancer	CRISPR-Cas9	completed	China	*ex vivo*	NCT03081715	^61^
PD-1 knockout engineered t cells for metastatic non-small cell lung cancer	CRISPR-Cas9	active, not recruiting	China	*ex vivo*	NCT02793856	^62^
Therapeutic vaccine plus PD-1 knockout in prostate cancer treatment	CRISPR-Cas9	recruiting	China	*ex vivo*	NCT03525652	^63^
PD-1 knockout EBV-CTLs for advanced stage Epstein-Barr virus (EBV) associated malignancies	CRISPR-Cas9	recruiting	China	*ex vivo*	NCT03044743	^64^
CD19 CAR and PD-1 knockout engineered T cells for CD19 positive malignant B cell derived leukemia and lymphoma	N.S.	not yet recruiting	China	*ex vivo*	NCT03298828	^82^
Study of PD-1 gene-knocked out mesothelin-directed CAR-T cells with the conditioning of PC in mesothelin positive multiple solid tumors	CRISPR-Cas9	recruiting	China	*ex vivo*	NCT03747965	^83^
CAR T and PD-1 knockout engineered T cells for esophageal cancer	N.S.	recruiting	China	*ex vivo*	NCT03706326	^84^
Anti-MUC1 CAR T cells and PD-1 knockout engineered T cells for NSCLC	N.S.	recruiting	China	*ex vivo*	NCT03525782	^85^
CRISPR (HPK1) edited CD19-specific CAR-T cells (XYF19 CAR-T Cells) for CD19^+^ leukemia or lymphoma	CRISPR-Cas9	recruiting	China	*ex vivo*	NCT04037566	^86^
Study of UCART19 in pediatric patients with relapsed/refractory B acute lymphoblastic leukemia (PALL)	TALEN	active, not recruiting	US/EU/UK	*ex vivo*	NCT02808442	^103^
Dose escalation study of UCART19 in adult patients with relapsed/refractory B cell acute lymphoblastic leukaemia (CALM)	TALEN	active, not recruiting	US/EU/UK/Japan	*ex vivo*	NCT02746952	^104^
Safety and efficacy of ALLO-501 anti-CD19 allogeneic CAR T cells in adults with relapsed/refractory large B cell or follicular lymphoma (ALPHA)	TALEN	recruiting	US	*ex vivo*	NCT03939026	^105^
Safety and efficacy of ALLO-715 BCMA allogenic CAR T cells in in adults with relapsed or refractory multiple myeloma (UNIVERSAL)	TALEN	recruiting	US	*ex vivo*	NCT04093596	^106^
A study to evaluate the long-term safety of patients with advanced lymphoid malignancies who have been previously administered with UCART19/ALLO-501	TALEN	enrolling by invitation	US/EU/UK/Japan	*ex vivo*	NCT02735083	^107^
A study evaluating UCART019 in patients with relapsed or refractory CD19^+^ leukemia and lymphoma	CRISPR-Cas9	recruiting	China	*ex vivo*	NCT03166878	^112^
A safety and efficacy study evaluating CTX110 in subjects with relapsed or refractory B cell malignancies	CRISPR-Cas9	recruiting	US/Australia/Germany	*ex vivo*	NCT04035434	^115^
A safety and efficacy study evaluating CTX120 in subjects with relapsed or refractory multiple myeloma	CRISPR-Cas9	recruiting	US/Australia	*ex vivo*	NCT04244656	^116^
CTA101 UCAR-T cell injection for treatment of relapsed or refractory CD19^+^ B cell acute lymphoblastic leukemia	CRISPR-Cas9	recruiting	China	*ex vivo*	NCT04154709	^117^
Phase I study of UCART22 in patients with relapsed or refractory CD22^+^ B cell acute lymphoblastic leukemia (BALLI-01)	TALEN	recruiting	US	*ex vivo*	NCT04150497	^118^
CTA101 in the treatment of relapsed or refractory diffuse large B cell lymphoma	CRISPR-Cas9	not yet recruiting	China	*ex vivo*	NCT04026100	^119^
A feasibility and safety study of universal dual specificity CD19 and CD20 or CD22 CAR-T cell immunotherapy for relapsed or refractory leukemia and lymphoma	CRISPR-Cas9	recruiting	China	*ex vivo*	NCT03398967	^120^
Study evaluating safety and efficacy of UCART123 in patients with acute myeloid leukemia (AMELI-01)	TALEN	recruiting	US	*ex vivo*	NCT03190278	^121^
Study evaluating safety and efficacy of UCART targeting CS1 in patients with relapsed/refractory multiple myeloma (MELANI-01)	TALEN	recruiting	US	*ex vivo*	NCT04142619	^122^
Anti-CD19 U-CAR-T cell therapy for B cell hematologic malignancies	N.S.	not yet recruiting	China	*ex vivo*	NCT04264039	^123^
Anti-CD7 U-CAR-T cell therapy for T/NK cell hematologic malignancies	N.S.	not yet recruiting	China	*ex vivo*	NCT04264078	^124^
Efficacy and safety evaluation of BCMA-UCART	N.S.	recruiting	China	*ex vivo*	NCT03752541	^125^
Safety and efficacy evaluation of CD19-UCART	N.S.	recruiting	China	*ex vivo*	NCT03229876	^126^
The clinical study of CD19 UCAR-T cells in patients with B cell acute lymphoblastic leukemia (B-ALL)	N.S.	recruiting	China	*ex vivo*	NCT04166838	^127^
NY-ESO-1-redirected CRISPR (TCRendo and PD1) edited t cells (NYCE T cells)	CRISPR-Cas9	terminated	US	*ex vivo*	NCT03399448	^133^
Study of CRISPR-Cas9 mediated PD-1 and TCR gene-knocked out mesothelin-directed CAR-T cells in patients with mesothelin positive multiple solid tumors	CRISPR-Cas9	recruiting	China	*ex vivo*	NCT03545815	^134^
Cell therapy for high risk T cell malignancies using CD7-specific CAR expressed on autologous T cells	CRISPR-Cas9	not yet recruiting	US	*ex vivo*	NCT03690011	^144^

**Cervical Cancer**						

Study of molecular-targeted therapy using zinc finger nuclease in cervical precancerous lesions	ZFN	N.S.	China	*in vivo*	NCT02800369	^160^
Study of targeted therapy using transcription activator-like effector nucleases in cervical precancerous lesions	TALEN	N.S.	China	*in vivo*	NCT03226470	^161^
A safety and efficacy study of TALEN and CRISPR/Cas9 in the treatment of HPV-related cervical intraepithelial neoplasia	CRISPR-Cas9 TALEN	N.S.	China	*in vivo*	NCT03057912	^162^

**HIV Infection and AIDS**						

Autologous T cells genetically modified at the CCR5 gene by zinc finger nucleases SB-728 for HIV	ZFN	completed	US	*ex vivo*	NCT00842634	^189^
Phase 1 dose escalation study of autologous t cells genetically modified at the CCR5 gene by zinc finger nucleases in HIV-infected patients	ZFN	completed	US	*ex vivo*	NCT01044654	^190^
Repeat doses of SB-728mR-T after cyclophosphamide conditioning in HIV-infected subjects on HAART	ZFN	completed	US	*ex vivo*	NCT02225665	^191^
A phase I study of T cells genetically modified at the CCR5 gene by zinc finger nucleases SB-728mR in HIV-infected patients	ZFN	completed	US	*ex vivo*	NCT02388594	^192^
Dose escalation study of cyclophosphamide in HIV-infected subjects on HAART receiving SB-728-T	ZFN	completed	US	*ex vivo*	NCT01543152	^193^
CCR5-modified CD4^+^ T cells for HIV infection (TRAILBLAZER)	ZFN	recruiting	US	*ex vivo*	NCT03666871	^194^
Study of autologous T cells genetically modified at the CCR5 gene by zinc finger nucleases in HIV-infected subjects	ZFN	completed	US	*ex vivo*	NCT01252641	^195^
Long-term follow-up of HIV subjects exposed to SB-728-T or SB-728mR-T	ZFN	enrolling by invitation	US	*ex vivo*	NCT04201782	^197^
Safety study of zinc finger nuclease CCR5-modified hematopoietic stem/progenitor cells in HIV-1 infected patients	ZFN	active, not recruiting	US	*ex vivo*	NCT02500849	^203^
Safety of transplantation of CRISPR CCR5 modified CD34^+^ cells in HIV-infected subjects with hematological malignances	CRISPR-Cas9	recruiting	China	*ex vivo*	NCT03164135	^204^
CD4 CAR+ ZFN-modified T cells in HIV therapy	ZFN	active, not recruiting	US	*ex vivo*	NCT03617198	^206^

**β-thalassemia and Sickle Cell Disease**						

A safety and efficacy study evaluating CTX001 in subjects with transfusion-dependent β-thalassemia	CRISPR-Cas9	recruiting	US/Canada/EU/UK	*ex vivo*	NCT03655678	^263^
A study to assess the safety, tolerability, and efficacy of ST-400 for treatment of transfusion-dependent beta-thalassemia (TDT)	ZFN	active, not recruiting	US	*ex vivo*	NCT03432364	^264^
A safety and efficacy study evaluating CTX001 in subjects with severe sickle cell disease	CRISPR-Cas9	recruiting	US/Canada/EU	*ex vivo*	NCT03745287	^265^
A study to assess the safety, tolerability, and efficacy of BIVV003 for autologous hematopoietic stem cell transplantation in patients with severe sickle cell disease (BIVV003)	ZFN	recruiting	US	*ex vivo*	NCT03653247	^266^
A long-term follow-up study in subjects who received CTX001	CRISPR-Cas9	enrolling by invitation	US/EU	*ex vivo*	NCT04208529	^267^
iHSCs with the gene correction of HBB intervent subjests with β-thalassemia mutations	CRISPR-Cas9	not yet recruiting	N.S.	*ex vivo*	NCT03728322	^280^

**Hemophilia**						

Ascending dose study of genome editing by zinc finger nuclease therapeutic SB-FIX in subjects with severe hemophilia B	ZFN	active, not recruiting	US	*in vivo*	NCT02695160	^289^

**Mucopolysaccharidoses**						

Ascending dose study of genome editing by the zinc finger nuclease (ZFN) therapeutic SB-318 in subjects with MPS I	ZFN	active, not recruiting	US	*in vivo*	NCT02702115	^319^
Ascending dose study of genome editing by the zinc finger nuclease (ZFN) therapeutic SB-913 in subjects with MPS II	ZFN	active, not recruiting	US	*in vivo*	NCT03041324	^320^

**Leber’s Congenital Amaurosis**						

Single ascending dose study in participants with LCA10	CRISPR-Cas9	recruiting	US	*in vivo*	NCT03872479	^329^

N.S., not specified.

### Gene Editing in Cancer Immunotherapy

Adoptive cell therapy (ACT) is a cellular form of cancer immunotherapy involving T cells with anti-tumor activity^45^ that are expanded *ex vivo*, *ex vivo* genetically engineered or not, and applied to the patient via the circulation. Three major types of lymphocytes are used in ACT: (1) tumor-infiltrating lymphocytes (TILs), which are T cells that are isolated from tumors; and peripheral blood T lymphocytes that are (2) selected for tumor reactivity and expanded *ex vivo* before reinfusion or (3) genetically modified *ex vivo* with a transgenic T cell receptor (tTCR) or a chimeric antigen receptor (CAR) to target tumor cells.^46^ ACT has been combined with *ex vivo* gene editing in a number of clinical trials, as discussed below.

#### Immune Checkpoint Knockout

Immune checkpoints are immune modulatory signals that can dampen the amplitude and quality of the immune response. Their physiological function is to prevent overstimulation of the immune system in order to maintain self-tolerance. A hallmark of cancer cells is their ability to exploit immune checkpoints to evade attack by the immune system. Cancer cells or their microenvironment can achieve this by activating immune checkpoints via overexpression of ligands or receptors that regulate the function of T cells.^47^^,^^48^ In this way, cancer cells escape immune surveillance. To exploit this property of cancer cells for anti-cancer therapy, monoclonal antibodies have been developed that block natural immune checkpoints (present on T cells) or their ligands (present on cancer cells or in their micro-environment). This has revolutionized the field of anti-cancer therapy.^49^ Examples include PD-1 and PD-L1 inhibitors, which have shown impressive results for treating different types of cancer at an advanced stage,^50^^,^^51^ especially melanoma.^52^ PD-1, encoded by the *PDCD1* gene, is a cell-surface receptor expressed on cytotoxic T cells that downregulates T cell activity upon interaction with its ligand PD-L1, which is overexpressed on malignant cells and cells in the tumor micro-environment.^48^ In spite of general good tolerability, systemic administration of immune checkpoint inhibitors can result in autoimmune phenomena, referred to as immune-related adverse events (IRAEs).^53^ IRAEs occur in up to 70% of patients receiving PD-1 and PD-L1 inhibitors^50^^,^^51^ and have been described in multiple organ systems. Steroids might be used to manage IRAEs, but the extent of interference with immunotherapy is unknown.^53^

**Figure 1:**
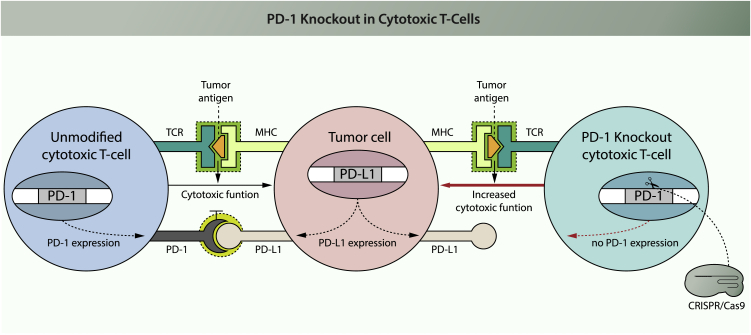
Effect of PD-1 Knockout in Cytotoxic T Cells Cytotoxic T cells are able to recognize tumor cells via the T cell receptor (TCR). This receptor recognizes an antigen that is presented on potential target cells by the MHC. Binding results in T cell activation through signal transduction. The activated T cell will expand and exert its cytotoxic effector function on target cells, thus inducing apoptosis. If the target cell expresses PD-L1, it can interact with PD-1 that is expressed on the surface of the T cell. This will lead to activation of PD-1, one of the immune checkpoint molecules, resulting in inhibition of the T cell’s cytotoxic activity. If *PD-1* is disrupted in the cytotoxic T cell, PD-L1 expressed from the tumor cell can no longer interact with the T cell and inhibition of T cell cytotoxicity is prevented. In this scenario, *PD-1* disruption prevents escape of tumor cells from attack by cytotoxic T cells. Red indicates the result of intervention.

Knocking out immune checkpoint molecules in tumor-specific T cells is a promising strategy for ACT to circumvent systemic effects of checkpoint inhibition (Figure 1). When applied to total T cells harvested from patients, knocking out immune checkpoint molecules should render these less susceptible to immune inhibitory signals upon reinfusion. However, such an approach involves a heterogeneous T cell population rather than tumor-specific T cells. One solution to this problem would be to increase tumor specificity of circulating T cells *in vitro* by exposure to tumor-associated antigens.^54^

Due to the impressive clinical results from checkpoint inhibitors and TILs to treat melanoma, this type of cancer was chosen in the initial preclinical studies on applying immune checkpoint knockout (KO) in ACT using *ex vivo* gene editing. Promising *in vitro* results were reported from co-cultures of human tumor-specific T cells in which PD-1 was disrupted with melanoma cell lines,^55^^,^^56^ and more recently by infusing PD-1 knockout T cells cells into mice that had been xenografted with human melanoma cells.^57^ An improved cytotoxic effect of tumor-specific T cells following PD-1 knockout was also reported in preclinical studies of other cancer models, such as in a cultured gastric cancer cell line,^56^ and in mice subcutaneously injected with either a fibrosarcoma cell line,^58^ a multiple myeloma (MM) cell line,^59^ or a liver cancer cell line.^60^ Academic hospitals have been recruiting patients in clinical trials to investigate autologous, PD-1 knocked out T cells for the treatment of multiple types of cancer, including solid tumors arising from the esophagus,^61^ lung,^62^ prostate,^63^ and Epstein-Barr-related neoplasms.^64^ The publicly provided information is scarce. Presumably, as described for preclinical studies, these T cells have been manipulated *ex vivo* to enhance their tumor specificity, but this has not been specified. Recently, the results for PD-1-edited T cells in metastatic lung carcinoma patients were published.^65^ Although no methods for increasing the tumor specificity of T cells was described, no severe adverse events were reported in 12 patients after a median follow-up time of 47.1 weeks. Despite the treatment, 10 patients progressed, and only 2 responded transiently. Although not designed to investigate the therapeutic effect, these results were somewhat disappointing and are possibly caused by inadequate levels of tumor-specific T cells.

Another method of generating tumor-specific T cell clones is the *ex vivo* expansion of T cells that are isolated from tumor tissue, so-called TILs. Although not yet clinically applied, PD-1 knockout in TILs has resulted preclinically in an improved anti-tumor effect *in vitro*^55^ and *in vivo*.^58^

Innate immune cells such as dendritic cells (DCs) and natural killer (NK) cells are also target cells for the development of immunotherapy against cancer.^66^ Importantly, NK cells have also been shown to express several immune checkpoint inhibitors.^67^ An example of recent preclinical developments is the knockout of the *NKp46* and *CIS* checkpoint genes in primary human NK cells.^68^^,^^69^ Although gene-edited innate immune cells have not yet reached clinical trials, these efforts illustrate the ongoing work that might promote their clinical development.

#### Immune Checkpoint Knockout in Genetically Engineered T Cells: tTCR-T and CAR-T cells

Besides the isolation of T cells with enhanced anti-tumor activity from patients, it is also possible to induce tumor specificity in T cells using genetic engineering (using viral transduction or gene editing). Such redirected T cells can be generated by forced expression of receptors with enhanced specificity for a tumor-associated antigen, such as tTCRs or CARs.^70^^,^^71^ tTCRs are transgenic forms of naturally occurring receptors isolated from tumor-specific T cells and depend on the major histocompatibility complex (MHC) for efficient antigen recognition.^72^ CARs are synthetic receptors that do not depend on MHC for efficient antigen binding.^73^ To avoid negative regulation by tumors, immune checkpoint inhibition (using antibodies) or knock out (using gene editing) are also worthwhile strategies in tTCR-T cells and CAR-T cells.

The concept of immune checkpoint knockout in redirected T cells has been demonstrated *in vitro* and *in vivo*, both for tTCR-T cells^74^ and CAR-T cells.^75^, ^76^, ^77^, ^78^, ^79^ Improved antitumor reactivity of redirected T cells after PD-1 disruption was observed in a range of preclinical cancer models, for example, models of melanoma,^74^ hepatocellular carcinoma,^75^ glioma,^76^^,^^79^ breast cancer,^77^ and erytroleukemia.^78^ In addition, encouraging clinical results have already been obtained by combining CAR-T cells with immune checkpoint inhibitors.^80^^,^^81^ Using gene editing, PD-1 knockout in CAR-T cells that were redirected against the B cell marker cluster of differentiation 19 (CD19)^82^ and membrane proteins mesothelin^83^ and MUC1,^84^^,^^85^ which are upregulated in a range of malignancies, are investigated in clinical trials for the treatment of B cell leukemia/lymphoma,^82^ multiple mesothelin-positive solid tumors (such as pancreatic cancer, cholangiocarcinoma cancer, and ovarian cancer),^83^ esophageal cancer,^84^ and lung cancer.^85^ One trial investigates the infusion of CAR-T cells carrying an HPK1 knockout in patients with relapsed or refractory CD19^+^ leukemia or lymphoma.^86^ HPK1 is a protein kinase that was found to suppress the anti-tumor response of T cells by attenuating TCR signaling.^87^ In addition, HPK1 exerts T cell inhibitory effects downstream of E prostanoid receptor activation by prostaglandin E2, a metabolic byproduct that is overproduced by cancers such as non-small-cell lung carcinomas.^88^^,^^89^ Mice with a kinase-dead HPK1 showed improved anti-tumor^89^^,^^90^ and antiviral responses.^90^

Disruption of other molecules with immunomodulatory effects in ACT has been performed in preclinical studies, but no clinical trials are currently open. For example, infusion of cytotoxic T cells in which the immune checkpoint gene *CTLA-4* was disrupted resulted in decreased tumor growth compared to infusion of non-edited counterparts in mice that were xenografted subcutaneously with bladder cancer cell lines^91^ or colon cancer cell lines.^92^ In addition, the anti-tumor effect of CAR-T cells against a human glioma cell line that was subcutaneously engrafted in mice was enhanced upon knockout of *DGK*,^93^ which encodes an intracellular enzyme that negatively regulates TCR signaling.^94^ In contrast, disruption of the immune checkpoint gene *LAG-3* in CAR-T cells did not result in an enhanced anti-tumor effect in mice subcutaneously engrafted with a human lymphoma cell line,^95^ suggesting that the choice of immune checkpoint gene is important to design an efficient treatment.

#### Universal ACT

So far, we discussed autologous T cell therapies. However, this is not always feasible for every patient.^96^ The establishment of universal, allogeneic ACT might be an attractive alternative, because such “off-the-shelf” therapy would overcome the high costs and experimental burden of manufacturing a custom-made autologous or histocompatibility leukocyte antigen (HLA)-matched allogeneic therapy for every patient. For such therapy, the risks of graft-versus-host disease (GvHD) and graft rejection by the patients’ immune system for universal ACT must be addressed. The strategies used involve knockout of the TCR to prevent GvHD, and knockout of human leukocyte antigen (HLA) genes to prevent graft rejection by the host immune system.^97^^,^^98^ Clinical studies and preclinical examples are discussed below.

*In vitro* studies showed that anti-CD19 CAR-T cells, which target B cells, tolerated ZFN-mediated knockout of the TCR, as assessed by cell proliferation and their ability to lyse target cells.^99^ In addition, *in vivo* application of such cells demonstrated an anti-leukemic response in mice that were intravenously injected with a lymphoma cell line that was similar or better compared to non-edited cells.^100^^,^^101^ The feasibility of clinical implementation of such a strategy was illustrated by a study in which two therapy-refractory pediatric patients with acute lymphoblastic leukemia (ALL) were treated with allogeneic anti-CD19 CAR-T cells from unselected donors^102^ that had been engineered *in vitro* using TALENs in two ways. First, expression of the endogenous αβ TCR was disrupted by targeting the constant region of the TCR α chain. Second, *CD52* was knocked out with the following rationale. CD52 is expressed on T cells, and anti-CD52 antibodies (alemtuzumab) are part of the conditioning regimen prior to allogeneic HSC transplantation to reduce the risk of graft rejection by the host’s lymphocytes. To prevent alemtuzumab from attacking anti-CD19 CAR-T cells, these cells were made resistant by knockout of *CD52*. Despite development of grade 2 GvHD in one of the patients, the results of this trial indicated an ongoing disease-free survival of the two patients of 12 and 18 months after the start of therapy.^102^ These results suggest that off-the-shelf allogeneic CAR-T cells therapy is feasible, and that adverse events such as GvHD are manageable. This exact strategy is adopted in clinical trials investigating universal CAR-T cells in pediatric or adult B cell ALL,^103^^,^^104^ B cell lymphoma,^105^ and MM patients.^106^ The long-term effects of two of these products are investigated in a separate trial.^107^

To reduce the risk of graft rejection by the host immune system, *HLA* genes have been disrupted in donor T cells.^108^, ^109^, ^110^, ^111^ Notably, CRISPR-Cas9-mediated triple KO of the T cell receptor α constant (*TRAC*) *locus*, an HLA complex gene (*B2M*), and an immune checkpoint gene (*PDCD1*) was used to potentiate the anti-tumor effect of CAR-T cells against multiple targets in mouse models, for example in mice intravenously injected with a B cell ALL cell line,^109^ in mice intraperitoneally injected with a lymphoma cell line,^110^ and in mice intracerebrally injected with a glioma cell line.^111^ In one active clinical trial both the endogenous TCR and HLA complex are knocked out in anti-CD19 CAR-T cells for treating of B cell leukemia and lymphoma.^112^

In another concept, a tumor-targeting CAR or tTCR is inserted into the *TRAC* locus using CRISPR-Cas9-mediated HDR. This yields two effects: knockout of the endogenous TCR, and knockin of the CAR/tTCR. In a preclinical study, a CD19-directed CAR was inserted into the *TRAC* locus in human T cells by HDR using CRISPR-Cas9.^113^ When these CAR-T cells were administered to a mouse model of ALL, an improved anti-leukemic response was observed that resulted in prolonged survival compared to conventionally generated CAR-T cells.^113^ A similar strategy proved feasible for inserting a tTCR directed against the immunogenic cancer antigen NY-ESO-1 in the *TRAC* locus.^114^ This strategy is adopted in two clinical trials for patients with B cell malignancies^115^ or MM,^116^ in which the endogenous TCR is disrupted by knockin of an anti-CD19 or anti-BCMA CAR in the TCR locus of allogeneic T cells, respectively. In addition, the HLA complex is disrupted by knockout of the B*2M* gene.

Additional clinical studies are planned, in which infusion of universal CAR-T cells (engineered using TALENs or CRISPR-Cas9) will be investigated for the treatment of B cell ALL or lymphoma,^117^, ^118^, ^119^, ^120^ acute myeloid leukemia (AML),^121^ and multiple myeloma.^122^ No molecular details are provided for these trials. Five more clinical trials are active or planned that will investigate universal CAR-T cells in hematological malignancies, but no information on the applied gene editing platform has been provided.^123^, ^124^, ^125^, ^126^, ^127^

A challenging application in one of the aforementioned trials is the treatment of AML with ACT, because molecular targets of leukemic cells in AML are also expressed in HSCs. As a result, ACT will attack the host’s HSCs and impair hematopoiesis.^128^ Indeed, severe myelotoxicity, leading to prolonged pancytopenia, was seen in preclinical studies using CAR-T cells directed at CD33^129^ and CD123.^130^ One possible strategy to circumvent this problem would be to co-transplant HSCs in which the target molecule is knocked out together with the CAR-T cells. As the CAR-T cells will attack the leukemic cells and unmodified recipient HSCs, the gene-edited donor HSCs will not be targeted anymore and will repopulate the bone marrow. This strategy has been proven feasible in a mouse model for AML, in which anti-CD33 CAR-T cells along with CD33-edited HSCs were used.^131^ The leukemic cells responded to anti-CD33 CAR-T cell treatment, while myelotoxicity was selectively mitigated in mice transplanted with CD33-edited HSCs. An ongoing clinical trial investigates universal CAR-T cells in refractory or relapsed AML, but it does not include a method to mitigate the possible myelotoxic effect of CAR-T cells.^121^

#### Endogenous TCR Knockout in Autologous ACT

Above we described the knockout of the endogenous TCR in allogeneic ACT products to prevent GvHD. However, there is also a rationale for knocking out endogenous TCR components in autologous ACT. The reason for this is that the endogenous TCR can interfere with the function of the tTCR/CAR, either by competing for cell surface expression, or by dimerization to form a novel hybrid compound TCR that might cause autoimmune reactions.^132^ Knockout of endogenous TCR components in autologous ACT cells is therefore adopted in two clinical trials with either tTCR-T cells redirected against NY-ESO-1 (in MM, melanoma, or subtypes of sarcoma)^133^ or CAR-T cells redirected against mesothelin (in any mesothelin-positive solid tumor).^134^ PD-1 is also knocked out in the tTCR-T cells and CAR-T cells in these trials. Initial results of the first trial have been published, and they indicated no major adverse events in the three patients that were included.^135^ The patients suffered from advanced refractory malignancies, and the response to therapy was variable: one patient did not respond and died, while two patients showed initial disease stabilization, followed by disease progression after 30 or 100 days. Responses to follow-up treatment in these two patients were variable. Interestingly, the authors reported a relatively long half-life of tTCR-T cells at an average of 83.9 days. As other studies reported a half-life of roughly 1 week of non-edited NY-ESO-1 tTCR-T cells,^136^, ^137^, ^138^ the knockout of PD-1 and/or endogenous TCR components might have contributed to a slower decay of the tTCR-T cells.

#### A Special Case: ACT for T Cell Malignancies

**Figure 2:**
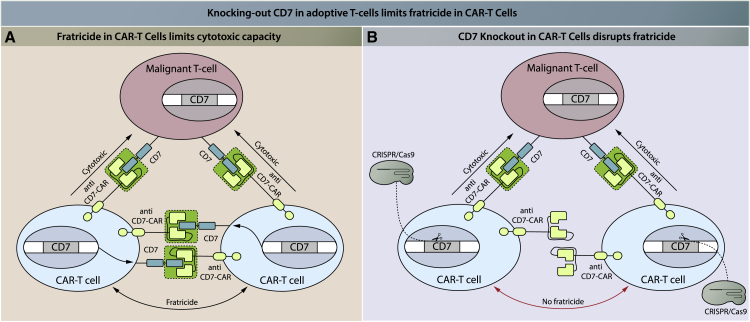
CD7 Knockout in Anti-CD7 CAR-T Cells Prevents Fratricide (A) Anti-CD7 CAR-T cells recognize the CD7 antigen on (malignant) T cells via their chimeric antigen receptor, which triggers the CAR-T cell cytotoxic function and thus results in lysis of the target cell. CD7 is expressed on the surface of all T cells. As CAR-T cells also express CD7, CAR-T cells will recognize other CAR-T cells and lyse these, which is termed fratricide. (B) The gene encoding CD7 can be knocked out in anti-CD7 CAR-T cells, for example by CRISPR-Cas9. Without CD7, these CAR-T cells will not be recognized and lysed by other anti-CD7 CAR-T cells, thus preventing fratricide. Red indicates the result of intervention.

It is particularly challenging to design an effective ACT using T cells for T cell malignancies. T cells should target molecules that are preferably expressed by malignant T cells but not by normal T cells. The difficulty in finding specific targets in malignant T cells results in self-destruction of tTCR-T cells or CAR-T cells cells used in ACT.^139^ This process, called fratricide, can interfere with ACT efficacy and has been observed in both CAR-T cells^140^ and transgenic TCR-T cells.^141^ One possible solution to this problem is to knockout the target molecule in the adoptive T cells by gene editing. In this way, transgenic TCR-T or CAR-T cells will recognize and attack malignant T cells, but not each other. This strategy has been proven effective in circumventing fratricide in a preclinical setting,^142^^,^^143^ and it is currently applied in a clinical trial applied to CD7. CD7 is expressed on the cell surface of T cells, and in this trial anti-CD7 CAR-T cells are tested for the treatment of T cell leukemia/lymphoma. To prevent fratricide, CD7 was knocked out in CAR-T cells using CRISPR-Cas9 (Figure 2).^144^ In addition, one previously mentioned clinical trial investigates universal anti-CD7 CAR-T cells in T cell malignancies, but knockout of CD7 in the CAR-T cells has not been mentioned.^124^

### Gene Editing in Viral Infection

#### Cervical Cancer

Cervical cancer is the third most prevalent type of cancer in women worldwide.^145^ The most contributing etiological factor is human papillomavirus (HPV) infection via sexual intercourse, especially serotypes HPV-16 and HPV-18. Most HPV infections are cleared by the host immune system, but persistent infections can give rise to malignant transformation. Several vaccines have been developed for primary prevention of cervix carcinoma, with varying levels of population coverage worldwide.^146^ Premalignant lesions are treated by local excision, while therapeutic modalities for invasive cervix carcinoma are dependent on the cancer stage and include surgery, radiotherapy, and chemotherapy.^147^ In spite of these preventive and curative modalities, survival rates of cervical cancer range from 93% at early disease stage to 15% at disseminated disease stage.^148^ New treatment modalities are crucial to increase survival rates of cervix carcinoma.

One such recent advance is RNA interference (RNAi)-mediated knockdown of the viral oncogenes E6 and E7, as these have been identified to drive and sustain HPV-related carcinogenesis.^149^ In multiple studies, knockdown of E6 and E7 resulted in increased cell death in HPV-positive cell lines.^150^^,^^151^ However, multiple obstacles, such as the occurrence of escape mechanisms and insufficient efficiency, have prohibited RNA-based strategies from entering clinical trials so far.^152^ Guided gene knockout might partially overcome these limitations. First of all, RNAi only lowers target gene expression, whereas gene editing can completely disrupt or delete a gene, leaving no room for residual gene expression. Mutation of the target region, a known escape mechanism of RNA viruses, as observed in studies using RNAi-mediated knockdown, likely still applies to knockout strategies using gene editing. Another escape mechanism, which is expression of viral suppressors of RNAi, is expected not to apply to gene editing.^153^ Investigating viral escape from strategies involving gene editing in cervical cancer caused by HPV will be an important aspect in future research. As is true for any cancer, it will be important to start treatment at the earliest stage possible and to use treatments that are highly efficient.

Gene editing for treating HPV infection has focused on *E6* and *E7*. It is generally appealing to target viral genes, because these are exogenous sequences, reducing the chances of unintended off-target events in endogenous genes. Successful knockout of *E6* and *E7* genes has been achieved via ZFNs,^154^ TALENs,^155^^,^^156^ and CRISPR-Cas9.^157^, ^158^, ^159^ The *in vitro* knockout of viral *E6* or *E7* sequences in HPV-infected cell line models caused inhibition of cell growth and cell viability, which is in line with results obtained from RNAi. In addition, gene-edited cells showed reduced capability to engraft in mice compared to unedited cells when transplanted subcutaneously.^154^^,^^155^^,^^157^ Results were consistent for targeting HPV-16 and HPV-18.^155^ Furthermore, *in vivo* gene editing with topically applied TALEN components using polymer-complexed T512 plasmids in K14-HPV16 transgenic mice, a model system for cervical HPV-16 infection, resulted in reduced viral DNA loads and a reversal of histological malignant abnormalities.^155^ As only the TALEN platform was topically applied *in vivo* in a cervical cancer mouse model,^155^ the effects of topically applied gene editing tools on cervical cancer could not be compared. Based on these results, multiple clinical trials have been designed to investigate gene editing of precancerous cervical lesions, directed at the HPV genome. These clinical trials apply either ZFN,^160^ TALEN,^161^^,^^162^ or CRISPR-Cas9^162^ gene editing platforms, which are administered either by topical gel or vaginal suppository.

In the future, topically applied gene editing tools might be investigated in combination with chemotherapy in metastasized cervix carcinoma. Preclinically, an additive anti-cancer effect of gene editing was already shown *in vitro* and *in vivo* in combination with cisplatin.^163^ In addition, the potential of HPV targeting extends beyond the treatment of cervix carcinoma, as HPV-related cancers include other anogenital cancers such as vulvar, vaginal, anal, and penile cancer, but also cancers in the head and neck region.^164^ In preclinical studies, CRISPR-Cas9-based strategies have been tested for treating other chronic viral infections, such as hepatitis B virus,^165^, ^166^, ^167^, ^168^, ^169^, ^170^, ^171^, ^172^ Epstein-Barr virus,^173^, ^174^, ^175^, ^176^ and human immunodeficiency virus (HIV) (see section below). As these viral infections affect distinct tissues and/or have distinct modes of action, these might need tailored strategies for delivery to the required target. An overview of these gene editing strategies is provided in a review by de Buhr and Lebbink.^177^

#### Gene Editing in HIV Infection and AIDS

HIV is a lentivirus that integrates its genome (after reverse transcription of its RNA into DNA) into the genome of host CD4^+^ T helper cells, forming a provirus. After the initial acute phase of infection, a pool of T cells remains latently infected. When the provirus becomes activated, host cells produce new viral particles and undergo cell death. This causes acquired immunodeficiency syndrome (AIDS) if the numbers of T helper cells drop to levels that are insufficient to effectively protect the host from infections or malignant transformations.^178^ Currently, HIV infections are treated by antiretroviral therapy (ART) to reduce the risk of progression to AIDS. However, ART needs to be taken life-long, requires adherence to the treatment regimen, and can have side effects and incomplete efficacy.^179^^,^^180^ Although no curative treatment has been found to date, there are two documented cases of HIV patients who have been cured from HIV infection. The first patient, known as the Berlin patient, received two HSC transplantations for AML, and has remained HIV-negative since.^181^^,^^182^ His donor harbored a homozygous *CCR5* Δ32/Δ32 loss-of-function allele, which had previously been known to impair infection of T cells by HIV-1.^183^ A similar second patient was identified recently.^184^ In addition, genetic association studies have shown that *CCR5Δ32* homozygotes are resistant to HIV infection, whereas heterozygotes display delayed progression of disease.^185^, ^186^, ^187^ It was therefore hypothesized that *ex vivo* disruption of *CCR5* in patient-derived T cells, followed by reinfusion, could mimic the curative outcome of the Berlin patient. *CCR5* was targeted by ZFNs in human primary CD4^+^ T cells, and biallelic gene disruption was achieved in 33% of modified cells *in vitro*.^188^ In an HIV infection mouse model, injection of *CCR5* KO T cells resulted in decreased viral load and an increased T cell population compared to wild-type T cells.^188^ Six out of a total of seven clinical trials assessing the infusion of autologous CD4^+^ *CCR5* knockout T cells using ZFNs have been completed,^189^, ^190^, ^191^, ^192^, ^193^, ^194^, ^195^ and results of one have been published.^196^ In the study of Tebas et al.,^196^ CD4^+^ CCR5 KO T cell infusion proved to be safe in HIV patients. In addition, levels of blood HIV DNA decreased in most patients, although the trial was not designed to measure efficacy. One clinical trial is currently investigating the long-term effects of *CCR5*-edited T cells.^197^

It is unclear how long engineered T cells can in principle protect against AIDS given their limited lifespan. Therefore, several groups are focusing on deleting *CCR5* in HSCs, as these have self-renewal capacity to remain present as stem cells and can give rise to all cells of the hematopoietic lineage.^198^ HSCs would for example also give rise to CD4^+^ myeloid cells, which are also susceptible to HIV infection.^199^ *CCR5* disruption by ZFNs was achieved in human CD34^+^ HSCs, and these cells were able to engraft in immunosuppressed or immunodeficient mice.^200^, ^201^, ^202^ In addition, infusion of *CCR5*-modified HSCs resulted in reduced plasma HIV levels in mouse models when compared to unmodified HSC infusions.^202^ Currently, two clinical trials are recruiting patients to test this strategy using either ZFN^203^ or CRISPR-Cas9.^204^

The previous strategies involve supplying patients with HIV-resistant cells to diminish the effect of HIV on the immune system. Alternatively, CAR-T cells that are redirected toward HIV-related proteins can be applied to actively attack T cells that are infected by the virus.^205^ Via gene editing, *CCR5* might be disrupted in the CAR-T cells to prevent HIV from infecting these cells. Multiple clinical trials are planned or ongoing for CAR-T cells as a treatment option for HIV. In one of those, ZFNs are applied to disrupt *CCR5* in CAR-T cells.^206^

*CCR5* disruption will not be efficacious in all patients, since CCR5 might be redundant for cell entry by certain HIV strains.^207^^,^^208^ Another disadvantage is the necessity of biallelic knockout of *CCR5* to efficiently impair viral reproduction.^198^^,^^209^ An alternative is disruption of the HIV genome itself, which may be especially attractive since this is not an endogenous sequence and may therefore be less susceptible to off-target effects. Targeted disruption of the HIV genome, however, faces the challenge of mutational escape. Another challenge is that HIV-1 forms a stable reservoir in resting CD4^+^ T cells, which sustains the disease and causes the residual viremia in patients undergoing ART.^210^ If the latent reservoir could be directly targeted or activated, HIV infection could possibly be cured without the requirement of myeloablative therapy and subsequent HSC transplantation. Multiple proof-of-principle studies have shown the feasibility of targeting HIV genomic sequences in infected cells *in vitro*,^211^, ^212^, ^213^, ^214^, ^215^, ^216^, ^217^, ^218^, ^219^ but the problems of mutational escape and targeting the HIV latent reservoir have not been solved to date.^220^

Alternatively, the strategies mentioned above could be realized via RNAi. *CCR5* knockdown by short hairpin RNA (shRNA) in HSCs or T cells has been readily tested in preclinical studies and is the subject of a phase I/II clinical trial.^221^ Targeting of HIV transcripts by RNAi has also been tested preclinically.^221^ Besides mutational esacape mentioned above, RNAi faces the additional challenge of transcriptional upregulation of the target in response to knockdown.^222^

Exciting preclinical studies have shown the feasibility for applying gene editing to the engineering of B cells that produce antibodies specific to a number of viruses, including Rous sarcoma virus (RSV), influenza virus, Epstein-Barr virus (EBV), or HIV, all of which are viruses for which there is to date no vaccine available. In the example of HIV, broad neutralizing antibodies (bNAbs) have been detected in a small number of infected individuals at ∼1–3 years after infection.^223^ These NAbs protect against HIV infection. Primary human B cells have been successfully engineered using CRISPR-Cas9 to produce NAbs against HIV,^224^ and a proof of principle using engineered mouse B cells provided protection against infection with RSV.^225^

### Gene Editing in Hematological Disorders

#### β-thalassemia and Sickle Cell Disease

β-thalassemia is an autosomal recessive disease with more than 200 known disease-associated variants in the gene coding for the hemoglobin β chain (*HBB*), resulting in a clinically variable phenotype. All of these variants cause reduced or abolished translation of the HBB protein.^226^ Approximately 98% of total adult hemoglobin is composed of hemoglobin A (HbA), which is formed by two β-globin subunits bound to two α-globin subunits.^227^ Reduced expression of the β-globin subunit results in a relative excess of the α-globin subunit, resulting in precipitation of the α-globin subunit in erythroblasts and erythrocytes. This ultimately leads to impaired erythropoiesis and hemolysis, and thus anemia.

Treatment of β-thalassemia depends on life-long supportive measures, of which blood transfusion is the main component. β-Thalassemia patients either have transfusion-dependent thalassemia (TDT) or non-TDT (NTDT).^228^ TDT patients require life-long blood transfusions for survival, starting at an average age of 2 years for every 2–5 weeks, while NTDT patients need blood transfusions only occasionally or for limited periods of time.^228^ Regular transfusions place patients at risk of blood-borne infections, iron overload, and transfusion reactions.^229^ In addition, 80% of TDT patients develop long-term complications.^230^ Although long-term complications due to iron overload result in decreased longevity, a life expectancy of over 50 years of age has been estimated.^231^ Recurrent therapy, adverse events, and complications also negatively impact patients quality of life. Furthermore, treatment of β-thalassemia patients with iron chelation therapy is essential to reduce iron overload, but it results in considerable additional costs. In addition, through alloimmunization, it becomes increasingly challenging to find eligible blood products.^229^ The only curative therapy to date is allogeneic HSC transplantation, provided that a suitable donor is available. An HLA-matched sibling donor is available in about 30% of cases.^232^ For the remaining patients an unrelated HLA-matched donor should be considered, which approaches success rates of sibling donors. However, for 20%–30% of patients needing an HSC transplantation (without considering the underlying disease), no optimal unrelated HLA-matched donor can be found even with the extensive donor registries that have been established in Europe.^233^ For 5% of patients, no donor could be identified at all. The alternative of cord blood transplantation from unrelated donors, for which HLA matching is less stringent, is less favorable due to higher rates of graft failure.^234^ Haploidentical, or half-matched (e.g., parents or children), stem cell transplantation seems inferior to HLA-matched unrelated transplantation due to delayed restoration of the immune system, although experience is limited.^232^ Between 2000 and 2010, the European Society for Blood and Bone Marrow Transplantation Hemoglobinopathy Registry reported treatment outcomes for all HSC transplantations, showing a 2-year event-free survival rate of more than 80% in TDT patients. However, this study also revealed a 12% overall mortality within 2 years after allogeneic HSC transplantation and the required (myeloablative) conditioning. In addition, 10% of patients developed severe acute GvHD, and about 15% of patients developed chronic GvHD.^235^

In sickle cell disease (SCD), the β-globin subunit in HbA carries a point variant that results in the formation of an aberrant form termed hemoglobin S. The *HBB* p.Glu6Val variant in combination with the same or a second *HBB* disease-associated variant on the second allele leads to SCD, in which erythrocytes are malformed, resulting in chronic hemolytic anemia. The malformed erythrocytes can cause acute ischemia throughout the body due to obstruction of blood vessels, leading to (severe) pain, organ failure, and severe acute vaso-occlusive complications such as acute chest syndrome or stroke,^236^ which can be treated by exchange transfusion.^237^ With this therapy, the patients’ blood is exchanged with donor blood to lower the percentage of sickle cells. Chronic transfusions are performed in patients with a history of stroke to prevent new cerebral ischemic events.^237^ Possible complications of frequent transfusions have been described previously. Frequently hospitalized patients, for example due to acute chest syndrome or the need for intravenous analgesics in the management of acute pain, are treated with hydroxyurea. These treatments, hospital admissions, acute complications, and many more chronic complications result in reduced life quality of patients.^237^ As in β-thalassemia, allogeneic HSC transplantation is the only cure for SCD. Although HSC transplantation with a product of a related HLA-matched donor seems successful in most cases, severe complications as described previously are also seen in SCD.^238^ Recent improvements in conditioning regimens have led to reduced intensity treatment without short-term GvHD, but serious adverse events still occurred.^239^^,^^240^ The experience with other HSC transplantation sources is scarce in SCD, but it seems inferior to related HLA-matched donors.^238^^,^^241^

Curative options that are less toxic than allogeneic HSC transplantation are required for both β-thalassemia and SCD. As gene therapy allows the engineering of autologous stem cells, the need for a donor would be bypassed. Importantly, transfusion of autologous rather than allogeneic stem cells strongly reduces the HSC transplantation-related toxicity.^242^ Reports of gene therapy using lentiviral vectors to add a healthy *HBB* copy to HSCs *in vitro* for reinfusion purposes have been published for β-thalassemia^243^ and SCD,^244^ and the first promising (interim) results of clinical trials have been reported.^245^^,^^246^ As the graft must replenish the hematopoietic system through rapid cell division, an integrative vector, such as lentiviral vectors, is required. Although γ-retroviral vectors used in the past gave rise to leukemia through insertional mutagenesis,^247^ currently used third-generation self-inactivating lentiviruses have an improved safety profile and have been used without adverse events in several clinical trials up to 7 years follow-up.^248^, ^249^, ^250^, ^251^, ^252^, ^253^, ^254^, ^255^ Because lentiviral transduction is highly efficient, it provides a strong competitor for gene editing approaches in strategies involving overexpression of transgenes.

**Figure 3:**
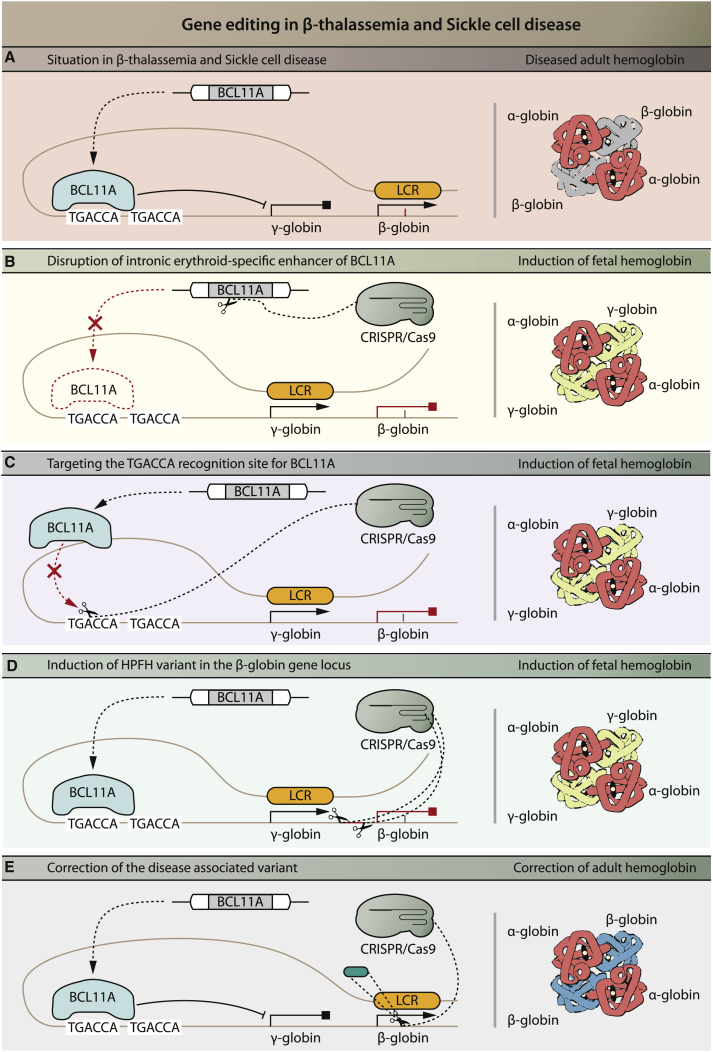
Gene Editing Strategies in β-Thalassemia and Sickle Cell Disease (A) Situation in β-thalassemia and sickle cell disease. The locus control region (LCR) loops to the β-globin gene and β-globin is expressed; however, due to a disease-associated variant in the β-globin gene there is insufficient expression (β-thalassemia) or malformed (sickle cell disease) β-globin. The transcriptional repressor BCL11A recognizes the first TGACCA binding sequence, which leads to inhibition of expression of fetal-specific γ-globin. (B) In one strategy, CRISPR-Cas9 or ZFNs (not shown) are used for targeted disruption of the GATAA motif in the intronic erythroid-specific enhancer of *BCL11A*, which will result in disruption of *BCL11A* expression during erythropoiesis and consequently relief of inhibition of γ-globin expression. γ-Globin will substitute for the lack of β-globin to form functional hemoglobin: HbF. (C) In a related strategy, the TGACCA recognition site for BCL11A is disrupted using CRISPR-Cas9 or ZFNs (not shown). BLC11A remains expressed but cannot bind to the recognition site to inhibit the γ-globin expression, resulting in relief of inhibition of γ-globin expression. (D) In another scenario, the β-globin promotor sequence is disrupted using CRISPR-Cas9, leading to a loss of binding sites for proteins that repress expression of γ-globin and subsequent induction of γ-globin expression. (E) Finally, the disease-associated variant can be precisely corrected using CRISPR-Cas9. While strategies in (B), (C), and (D) will lead to the induction of fetal hemoglobin, the strategy in (E) will lead to production of adult hemoglobin. Red indicates the result of intervention.

The main strategy under current investigation for clinical application of gene editing is the induction of endogenous expression of fetal hemoglobin (HbF). This originated from the observation that co-inheritance of hereditary persistence of HbF (HPFH), a benign condition, reduces symptoms of SCD and β-thalassemia.^227^ The situation in SCD and β-thalassemia is depicted in Figure 3A. In HPFH, HbF protein production continues into adulthood, whereas under normal physiological conditions production shifts to adult hemoglobin after birth. HbF protein contains two subunits of α-globin and γ-globin each, the latter of which are translated from the *HBG* gene. Persistent HbF expression in HPFH compensates for the reduced production of HbA in β-thalassemia patients. There is a difference in HbF protein levels among β-thalassemia patients, and this has been linked to several genetic variants, with single nucleotide variants (SNVs) in the *BCL11A* gene correlating most strongly with HbF expression.^256^ Reduced BCL11A protein expression is correlated with increased HbF protein expression, likely because BCL11A suppresses HbF expression by binding directly to the *HBG* promoter.^257^^,^^258^ *BCL11A* null mice were shown to be unable to downregulate murine embryonic globin in erythrocytes, demonstrating the essential role of BCL11A in repression of HbF expression during development.^259^ However, BCL11A knockdown by gene disruption in HSCs results in impaired engraftment of HSC in mice, illustrating that knockout of BCL11A itself is not a feasible strategy to treat β-thalassemia.^260^ As BCL11A expression during erythropoiesis is specifically regulated by the intronic erythroid-specific enhancer,^261^ disrupting this enhancer will result in BCL11A knockout during erythropoiesis, exclusively. This strategy was preclinically tested by using ZFN-mediated gene disruption of the GATAA element of the BCL11A erythroid-specific enhancer in HSCs (Figure 3B).^260^^,^^262^ These cells achieved robust long-term engraftment in mice and gave rise to erythroid cells with elevated HbF levels upon *ex vivo* culture of chimeric bone marrow.^260^ Multiple clinical trials are based on a strategy involving HSCs, of which the intronic erythroid-specific enhancer of BCL11A is disrupted *ex vivo* using CRISPR-Cas9 or ZFNs as a treatment for TDT^263^^,^^264^ or SCD^265^^,^^266^ patients. The long-term effects of infusing such cells are investigated in one clinical trial.^267^ Other strategies to increase HbF expression include disruption of the binding motif for BCL11A (and co-repressive proteins) within the HBG promoter sequence (Figure 3C)^257^^,^^268^^,^^269^ or the induction of a natural occurring variant termed the Sicilian HPFH disease-associated variant (Figure 3D).^270^ In the latter variant, the entire β-globin locus is deleted and the putative 3′ β-globin enhancer is brought in closer proximity to the γ-globin locus. These strategies have been explored preclinically, but have not (yet) reached clinical application.

Besides induction of HbF, other applications of gene editing techniques to treat β-thalassemia and SCD have been tested mainly in preclinical studies. Cai et al.^271^ showed an approach to correct various *HBB* disease-associated variants by inserting a cDNA sequence of exons 2 and 3 of the *HBB* gene downstream of *HBB* exon 1 *in vitro* using CRISPR-Cas9 in induced pluripotent stem cells (iPSCs). This strategy ensured expression of correct β-globin and prevented expression of the mutated variant in iPSC-derived erythrocytes. Other preclinical studies showed the (HDR-mediated) correction of a specific disease-associated variant in (iPSC-induced) HSCs to restore β-globin and thus HbA expression (Figure 3E).^272^, ^273^, ^274^, ^275^, ^276^, ^277^, ^278^, ^279^ One clinical trial implies to investigate the infusion of autologous, iPSC-induced HSCs with a directly gene-corrected version of the *HBB* gene in β-thalassemia.^280^ However, very limited information is provided for this trial and the exact strategy is not elucidated. Another preclinical strategy involves the *in vitro* knockout of α-globin^281^ to prevent its precipitation. No clinical trial has been reported that investigates this option.

#### Gene Editing in Hemophilia

Hemophilia A and B are congenital bleeding disorders caused by deficiencies in clotting factor VIII (FVIII) or IX (FIX), respectively. These diseases have a recessive X-linked inheritance pattern. Protein substitution therapy (PST) with recombinant clotting factor or protein derived from donor plasma is currently the main treatment for these patients.^282^ Despite the steep increase in life expectancy and the improved prevention of arthropathies due to articular bleedings after introduction of PST, this treatment has its drawbacks.^282^^,^^283^ As substituting a deficient protein is not curative, repeated administration is required and patients remain at risk of bleedings. In addition, costs related to PST are considerable.^282^ Insertion of a functional copy of the deficient gene in patient cells could potentially provide a long-term cure for hemophilia. To this end, *in vivo* gene therapy by viral vectors has been applied in multiple phase I clinical trials,^284^, ^285^, ^286^ as well as by *ex vivo* electroporation of fibroblasts that provided a source of FVIII after engraftment.^287^ Initial results observed in these clinical trials were disappointing.^282^ For lentiviral transduction, preclinical optimization of *ex vivo* HSC-mediated lentiviral gene therapy is paving the way for the first clinical studies.^282^ In spite of subclinical effects of targeting muscle cells by AAV vectors in hemophilia, promising clinical results have been obtained by the use of AAV vectors targeting liver cells.^282^ Transient liver toxicity and a temporary requirement for immunosuppressive therapy were drawbacks of this strategy.

Currently, gene editing strategies to target liver cells are also being explored for hemophilia. Sharma et al.^288^ achieved robust expression of human FVIII or FIX by integrating the cDNA of either gene into intron 1 of the albumin locus in primary hepatocytes *in vitro* and in hepatocytes of mice *in vivo* by using AAV-delivered ZFN-mediated gene editing. Despite the low *in vivo* genome editing efficiency, gene expression was achieved by placing the genes under the control of the highly active albumin promoter. This *in vivo* gene editing strategy is currently being investigated in hemophilia B patients in a clinical trial.^289^ A drawback for clinical implementation of such strategy is the long-term presence of active gene editing components in the liver of patients and the associated risk of damaging the genome by introducing double-stranded breaks at off-target loci. This is a serious concern, as the gene editing machinery delivered by AAV has an expected presence in the liver of several years, which significantly increases the chance for off-target effects to occur. This highlights the need for developing more transient ways to perform *in vivo* gene editing.

Other preclinical strategies that are under investigation include insertion of the transgene into the AAVS1 locus^290^^,^^291^ or in the native locus^292^, ^293^, ^294^, ^295^ and correction of disease-associated variants^296^, ^297^, ^298^ or large chromosomal rearrangements.^299^, ^300^, ^301^

For the clinical translation of gene editing in HSCs, a critical aspect is to maintain long-term engraftment capacity.^201^^,^^302^, ^303^, ^304^, ^305^ Similar to most other cells, HDR-mediated gene editing is challenging in HSCs, as these cells prefer the NHEJ pathway. In addition, it has been found that genetic manipulation of HSCs with gene editing or viral vectors can reduce their engraftment capacity. This has been found to be caused by activation of the DNA damage response pathway, resulting in activation of p53. Transient inhibition of p53 has been found to improve long-term engraftment of HSCs after gene editing.^344^ In addition, technical optimizations related to cell culture, delivery, and use of reagents have resulted in enhanced long-term engraftment of HSCs after gene editing in xenograft experiments involving transplantation of human HSCs into immunodeficient mice. The clinical testing of long-term engraftment of gene-edited HSCs in human patients needs further testing.

### Gene Editing in Metabolic Disorders

#### Mucopolysaccharidoses

Mucopolysaccharidoses (MPSs) are monogenic lysosomal storage diseases (LSDs) in which one of the enzymes involved in the lysosomal degradation of glycosaminoglycans (GAGs) is deficient. In MPS I and II, this concerns the α-l-iduronidase (IDUA) and iduronate-2-sulfatase (IDS) enzymes, respectively. Patients suffer from multisystemic symptoms and reduced life expectancy that can vary depending on the type of MPS and the severity of the disease-associated variant.^306^ The currently available treatment for MPS I, MPS II, MPS IVA, MPS VI, and MPS VII is enzyme replacement therapy (ERT), in which recombinant enzyme is administered intravenously. Drawbacks of ERT include the non-curative nature of the treatment, the requirement of repeated intravenous infusions, high costs, and ineffectiveness in treating symptoms in bone, cartilage, heart valves, and the central nervous system.^307^^,^^308^ In addition, generation of antibodies against the recombinant enzyme can interfere with the efficacy of ERT.^307^ HSC transplantation is currently applied to treat MPS I.^309^ This relies on the principle that lysosomal enzymes are secreted and can be taken up by target cells via the cation-independent mannose 6-phosphate receptor (CI-M6PR). In HSC transplantation, HSCs and their progeny secrete the enzyme into the circulation and provide a continuous source of ERT. In the case of MPS I, the level of secretion and reuptake provides partial efficacy in target organs. However, HSC transplantation depends on the availability of HLA-matched donors and can have severe adverse events such as GvHD, infection, and even death, as described before.^306^ In addition, the therapeutic effect on the skeletal abnormalities and neurological symptoms is limited, and for many other LSDs, endogenous expression levels in HSCs are insufficient to treat target organs. Therefore, overexpression by *ex vivo* lentiviral transduction or gene editing provides (additional) therapeutic efficacy. For MPS I, liposome-mediated delivery of CRISPR-Cas9 has been successfully applied *in vivo* and resulted in increased IDUA expression in newborn MPS I mice.^310^ Alternatively, direct gene addition using AAV vectors (without gene editing) has been shown feasible in preclinical studies for several MPS types.^311^, ^312^, ^313^, ^314^, ^315^, ^316^ This strategy is being investigated in multiple clinical trials, and recent results using intracerebral delivery showed promising outcomes with respect to treating the neurological decline of MPS IIIB patients.^317^

Another approach, similar to the approach in hemophilia, is the site-specific integration of a transgene in the liver by *in vivo* genome editing following intravenous administration using AAV as the delivery method.^288^ Most efforts have been made on integrating transgenes into the albumin locus. Sharma et al.^288^ achieved ZFN-mediated insertion of IDUA and IDS *in vivo* into the albumin locus of healthy mice, resulting in detectable protein levels in liver lysates. More recently, ZFN-mediated insertion of human IDS in the albumin locus in murine liver *in vivo* was accompanied by a dose-dependent rise in circulating enzyme levels.^318^ This IDS insertion caused reduction of GAG levels in tissue and urine samples of MPS II mice. These results have led to clinical trials investigating the safety of ascending dose levels of AAV vectors containing components required for *in vivo* ZFN-mediated insertion of IDUA and IDS genes into the albumin locus of hepatocytes in the liver of MPS I patients^319^ and MPS II patients,^320^ respectively. The same drawbacks as in the hemophilia trial with respect to safety due to the potential introduction of double-stranded breaks in the liver at off-target locations in the genome apply here due to the long-term exposure of the patient to the uncontrolled activity of ZNF-mediated double-stranded breaks.

### Gene Editing in the Eye

#### Leber’s Congenital Amaurosis

Leber’s congenital amaurosis (LCA) is an inherited retinopathy in which severe visual impairment or blindness occurs within the first months of life.^321^ It is a genetically heterogeneous disease that can be caused by any of more than 20 mutated genes. Based on the genes involved and the ocular phenotypes, LCA is divided into 13 subtypes.^322^ Currently, there is no treatment for LCA. In clinical trials, it has already been shown that AAV-mediated gene transfer by subretinal injection resulted in improved visual parameters in patients with the LCA type LCA2, which is caused by variants in the *RPE6* gene.^323^, ^324^, ^325^, ^326^ Retinal dystrophy in LCA was (at least partially) reversed by the therapy. AAV-mediated gene therapy has also been applied to other congenital retinopathies.^327^

In addition to AAV-mediated gene transfer, gene editing is in development for retinopathies. For subtype LCA10, which is caused by variants in the *CEP290* gene,^321^^,^^328^ a clinical trial is currently open^329^ with the strategy outline below. Gene transfer via viral vectors (especially AAV) is problematic for *CEP290* due to the large gene size. *CEP290* encodes a protein that is essential for cilia, which are microtubule-based, hair-like extensions of cell membranes.^330^ In photoreceptor cells, cilia are highly specialized into cone- or rod-shaped segments that act as light sensors and signal transducers.^322^ In LCA10, *CEP920* disease-associated variants cause (peripheral) thickening of the retina by an unknown mechanism.^330^ The most common variant is the intronic variant IVS26, which results in the generation of a cryptic splice site that causes an abrogated protein product.^328^ Preclinical studies had shown that, using subretinal injections of AAV5 vectors containing the CRISPR-Cas9 gene editing machinery, it deletion of the cryptic splice site leads to restoration of canonical splicing and expression of wild-type protein.^331^^,^^332^ This concept is used in the ongoing clinical trial.^329^ Other preclinical studies are investigating gene editing strategies for other disease-associated variants in LCA and other retinopathies.^333^^,^^334^ However, long-term expression of CRISPR-Cas9 in the eye imposes safety risks, as discussed in approaches for *in vivo* gene editing in hemophilia and MPS I and II.

### Conclusions and Future Prospects

#### Disease-Specific Challenges

The challenges and opportunities of applying gene editing for the treatment of human disease depend in part on disease-specific aspects. In cancer immunotherapy, a major challenge is to specifically target cancer cells while leaving healthy cells unharmed. Targeting immune checkpoints with gene editing has been shown to be a promising strategy, but the clinical feasibility relies in part on the inherent problem of specificity: by inhibiting a general checkpoint with the aim to inhibit negative immune regulation, there is a risk of auto-immune-related side effects. Considering the life-threatening nature of cancer, this disadvantage may be acceptable if survival rates can be improved and increased toxicity is manageable. Other challenges include the viability of T cells that have been gene edited *ex vivo* to knock out immune checkpoint regulators. These cells do not need to be present life-long, but they should have sufficient viability in order to help eliminating cancer cells. If needed, repeated administration would be an option, but this will increase costs. The development of a universal ACT would be an elegant solution to the high costs of preparing autologous or HLA-matched allogeneic gene-edited T cells for each individual patient, although this approach has the risk of inducing GvHD.

Application of targeted knockout to viral infection such as HPV could provide a useful additional treatment option when it comes to treating the primary tumor. However, a high effciency of gene knockout is reqired to effectively reduce the tumor, and treating metastases is not yet possible due to the difficulty to reach target tissues and to eliminate the HPV virus in a safe and efficient manner outside the primary tumor. It might be an advantage to target viral sequences rather than endogenous genomic locations to reduce the risks of undesired genomic alterations as the result of gene editing. This could also be a potential advantage for strategies that eradicate HIV provirus from the genome. In the case of HIV, disease-specific challenges include the targeting of the dormant HIV reservoir, and to target HIV strains that do not depend on CCR5 for infection.

#### *Ex Vivo* Gene Editing

In both genetic disease and viral infection, promising strategies using *ex vivo* gene editing lie ahead for disorders that can be cured via the blood, including hematological disorders, lysosomal storage disorders, and HIV infection. The main reason for this is the feasibility to target blood cells such as HSCs or T cells *ex vivo* and to engraft autologous gene-modified cells back into patients. This approach relies on the long-standing experience with successful engraftment of HSCs, which has nowadays become a standard procedure with a very good safety profile. In addition, engrafted HSCs can provide a life-long treatment because they can self-renew to sustain the stem cell population and to differentiate into the hematopoietic lineage. Because prolonged *ex vivo* culture reduces engraftment potential and stem cell properties of HSCs, fast and efficient methods are required to make *ex vivo* gene editing of HSCs feasible for clinical implementation. It remains to be seen whether *ex vivo* gene editing for overexpressing proteins will be able to successfully compete with *ex vivo* lentiviral transduction of HSCs when it comes to clinical implementation, because lentiviral transduction is highly efficient, has been used more than 7 years without adverse events in several clinical trials, and could be more cost-effective.^248^, ^249^, ^250^, ^251^, ^252^, ^253^, ^254^, ^255^ Strategies that rely on NHEJ are inherently more efficient compared to the HDR-mediated insertion of transgenes, and these provide promising options for the treatment of HIV infection, by knocking out the CCR5 receptor, or some genetic disorders such as β-thalassemia and SCD, by knocking out regulatory elements required for BCL11A-mediated negative regulation of HbF expression.

Among the many other preclinical developments for using *ex vivo* gene-edited HSCs (not covered in this review), the primary immunodeficiency diseases (PIDs) represent a promising example.^335^^,^^336^ These patients usually benefit from allogeneic HSC transplantation from HLA-matched donors, but these are not always available, and allogeneic HSCs can induce GvHD. Autologous HSC transplantation following *ex vivo* gene therapy employing third-generation lentiviruses is ongoing in a number of clinical trials for Wiskott-Aldrich syndrome, ADA severe combined immunodeficiency (ADA-SCID), X-linked SCID, and chronic granulomatous disease (CGD). However, many PIDs involve genes with a timed and restricted expression pattern during development and require endogenous expression levels via the natural promoter rather than overexpression. Gene editing would be advantageous above lentiviral transduction in these cases, as it enables precise correction of an endogenous allele to maintain endogenous expression levels. There are currently no clinical trials for PIDs reported using gene editing, but promising preclinical developments may change this in the near future.

Other promising preclinical developments include the *ex vivo* gene editing of primary hepatocytes for metabolic disease of the liver. As a proof of concept, AAV-mediated delivery of CRISPR-Cas9 to freshly isolated mouse hepatocytes was used, followed by engraftment into the liver of a mouse model. This concept was applied to treat hereditary tyrosinemia in a mouse model to correct a point variant in the fumarylacetoacetate hydrolase gene using HDR.^337^ A major challenge for this approach is the limited engraftment capacity of hepatocytes in human liver. In cystic fibrosis, investigators are pursuing gene editing of stem cells derived from the airways with the ultimate goal of developing a gene-edited autologous airway stem cell transplantation.^338^ Mitochondrial diseases that are caused by disease-associated variants in mitochondrial DNA form an attractive target for gene editing.^339^^,^^340^ However, gene editing of mitochondrial DNA is even more challenging than nuclear DNA, and improvements are required before clinical applications can be considered in the near future.

In all of these possible applications, the *ex vivo* mode of gene editing ensures that a quality control can be performed prior to decision-making of engrafting cells into patients. Reliable methods to assess undesired genomic alterations are essential, and a shift from methods that rely on predictions toward unbiased methods will be required. Quality control should also include functional analysis of cellular transformation, because rare events that result in formation of tumorigenic cells will be very difficult to detect in population-based assays.

#### *In Vivo* Gene Editing

*In vivo* gene editing trials have already started for a number of disorders including lysosomal storage disorders, hemophilia, precancerous cervical lesions and LCA. This is despite the uncertainties of gene editing technology with respect to possible off-target effects. This is particularly important when gene editing technology is introduced in patients without ways for spatiotemporal control (i.e., means to confine gene editing to a short time and specific target tissue, for example by using suicide genes in DNA combined with tissue-specific delivery, or local administration of gene editing tools as RNA/protein rather than DNA), such as is the case in trials so far. This means that gene editing may continue for years inside the patient, which will increase the risk of undesired events with several orders of magnitude compared to *ex vivo* gene editing. For safe future clinical development, it will be important to develop ways that can control the activity of *in vivo* gene editing by including on and off switches to prevent the prolonged generation of DNA breaks or by providing the gene editing machinery in other ways than as DNA. In addition, targeting gene editing tools specifically to the cells of interest will further increase the safety by preventing unnecessary targeting events in irrelevant cell types.

These aspects will also guide ongoing preclinical efforts to develop treatments for human disease based on *in vivo* gene editing. Multiple preclinical developments in different fields are ongoing, and it is beyond the scope of this review to cover these. As examples we mention metabolic disorders that are amenable to correction via knockout of a gene in the metabolic pathway to enable redirecting of metabolism. For example, severe hereditary tyrosinemia type I was successfully redirected to a more begin tyrosinemia type III form by deletion of the upstream metabolic enzyme hydroxyphenylpyruvate dioxygenase in the liver. The method applied involved intravenous injection of DNA encoding Cas9 and sgRNAs in the mouse, which transfected the liver more efficiently compared to other organs.^341^ The same gene was also targeted in as study on *in utero* correction of hereditary tyrosinemia type I using injection of an adenovirus expressing a base editor and sgRNA into mouse fetuses via the vitelline vein. In the same study, in utero knockout of PCSK9 was achieved with the aim to lower cholesterol levels and the risk of coronary heart disease in wild-type mice.

Precise correction of a point variant *in vivo* has been demonstrated for example in a mouse model for phenylketonuria (PKU) using base editors that were delivered by intravenous injection and that were expressed via a liver-specific promoter.^342^ Gene editing is even applied in preclinical research to increase the fitness of pig organs for future xenotransplantation into humans. By knockout of genes that activate an immune response and retroviral elements, the aims are to generate organs with reduced hazard of graft rejection and xenozoonosis (an infectious disease transmitted from animal to human), respectively.^343^ The ultimate goal of these efforts is to overcome the shortage of human organs such as kidneys, hearts, livers, and lungs for transplantation.

#### Keeping Up with Technological Developments

Finally, it will be important to educate the various stakeholders, including clinicians, patients, and regulatory institutions. The technology for gene editing is moving so fast that it is difficult to cope with all of the developments and their potential benefits and risks. Clinicians need to be educated in order to allow them to judge the feasibility of a clinical trial and whether they are willing to expose their patients to the novel treatment. Patients rely largely on the information that is provided by their treating physician. The prospect of a “cure” via gene repair may be tempting for a patient, and therefore providing balanced and fair information by the physician on the possible benefits and risks provides an essential ingredient for decision-making. The same arguments apply to regulatory institutions, as these will approve or decline clinical protocols and finally market authorization. While the scientific developments in the field of gene editing are continuing with dazzling speed, it will be important to provide education in the field and to closely monitor and regulate clinical developments.

In this review, we compiled all current clinical applications of gene editing and explained the rationale for the underlying strategies. In addition, we summarized preclinical studies that preceded clinical trials and provided examples of preclinical work that might be translated in a clinical setting in the future. As most other reviews focus on specific areas involving gene editing applications, we envision that centralized information on gene therapies will increase awareness of clinicians and researchers in the field of gene therapy outside their specific field of interest, and that this might catalyze new developments. We propose that clinical applications of gene editing in general will be documented in an accessible and transparent manner. We hope that this review precedes the discussion of a central database that includes relevant information of the clinical studies applying gene editing, as well as the underlying considerations with respect to the mechanism of action, safety, and expected results. Ideally, this information should be contributed by investigators involved in these clinical trials, peer-reviewed by experts in the field, and made publicly available prior to the start of such trials. Preferably, an analysis of risks and benefits of gene editing for a specific disease in the context of current treatments should be included, contributing to discussions on technical and ethical aspects of the applications. Such efforts should contribute to increasing transparency and help to inform stakeholders that are involved in clinical trials involving gene editing.

## Author Contributions

M.P.T.E., P.H.-H., M.B., and W.W.W.P.P. conceptualized this review, performed literature studies, and wrote the manuscript. All authors interpreted the contents and approved the final manuscript.

## Conflicts of Interest

A.T.v.d.P. has provided consulting services for various industries in the field of Pompe disease under an agreement between these industries and Erasmus MC, Rotterdam, the Netherlands. The remaining authors declare no competing interests.

## References

[1] Colella P., Ronzitti G., Mingozzi F. (2017). Emerging issues in AAV-mediated *in vivo* gene therapy. Mol. Ther. Methods Clin. Dev..

[2] Naldini L. (2015). Gene therapy returns to centre stage. Nature.

[3] Shirley J.L., de Jong Y.P., Terhorst C., Herzog R.W. (2020). Immune responses to viral gene therapy vectors. Mol. Ther..

[4] Rainov N.G., Ren H. (2003). Clinical trials with retrovirus mediated gene therapy—what have we learned?. J. Neurooncol..

[5] Ronzitti G., Gross D.A., Mingozzi F. (2020). Human immune responses to adeno-associated virus (AAV) vectors. Front. Immunol..

[6] Gaj T., Gersbach C.A., Barbas C.F. (2013). ZFN, TALEN, and CRISPR/Cas-based methods for genome engineering. Trends Biotechnol..

[7] Broeders M., Herrero-Hernandez P., Ernst M.P.T., van der Ploeg A.T., Pijnappel W.W.M.P. (2020). Sharpening the molecular scissors: advances in gene-editing technology. iScience.

[8] Komor A.C., Kim Y.B., Packer M.S., Zuris J.A., Liu D.R. (2016). Programmable editing of a target base in genomic DNA without double-stranded DNA cleavage. Nature.

[9] Gaudelli N.M., Komor A.C., Rees H.A., Packer M.S., Badran A.H., Bryson D.I., Liu D.R. (2017). Programmable base editing of A•T to G•C in genomic DNA without DNA cleavage. Nature.

[10] Sadelain M., Papapetrou E.P., Bushman F.D. (2011). Safe harbours for the integration of new DNA in the human genome. Nat. Rev. Cancer.

[11] van der Wal E., Herrero-Hernandez P., Wan R., Broeders M., In ’t Groen S.L.M., van Gestel T.J.M., van IJcken W.F.J., Cheung T.H., van der Ploeg A.T., Schaaf G.J., Pijnappel W.W.M.P. (2018). Large-scale expansion of human iPSC-derived skeletal muscle cells for disease modeling and cell-based therapeutic strategies. Stem Cell Reports.

[12] Hsu P.D., Scott D.A., Weinstein J.A., Ran F.A., Konermann S., Agarwala V., Li Y., Fine E.J., Wu X., Shalem O. (2013). DNA targeting specificity of RNA-guided Cas9 nucleases. Nat. Biotechnol..

[13] Kosicki M., Tomberg K., Bradley A. (2018). Repair of double-strand breaks induced by CRISPR-Cas9 leads to large deletions and complex rearrangements. Nat. Biotechnol..

[14] Cornu T.I., Mussolino C., Cathomen T. (2017). Refining strategies to translate genome editing to the clinic. Nat. Med..

[15] Kim D., Luk K., Wolfe S.A., Kim J.S. (2019). Evaluating and enhancing target specificity of gene-editing nucleases and deaminases. Annu. Rev. Biochem..

[16] Manghwar H., Li B., Ding X., Hussain A., Lindsey K., Zhang X., Jin S. (2020). CRISPR/Cas systems in genome editing: methodologies and tools for sgRNA design, off-target evaluation, and strategies to mitigate off-target effects. Adv. Sci. (Weinh.).

[17] Pattanayak V., Guilinger J.P., Liu D.R. (2014). Determining the specificities of TALENs, Cas9, and other genome-editing enzymes. Methods Enzymol..

[18] Yin H., Kauffman K.J., Anderson D.G. (2017). Delivery technologies for genome editing. Nat. Rev. Drug Discov..

[19] Lino C.A., Harper J.C., Carney J.P., Timlin J.A. (2018). Delivering CRISPR: a review of the challenges and approaches. Drug Deliv..

[20] Tong S., Moyo B., Lee C.M., Leong K., Bao G. (2019). Engineered materials for in vivo delivery of genome-editing machinery. Nat. Rev. Mater..

[21] Li A., Tanner M.R., Lee C.M., Hurley A.E., De Giorgi M., Jarrett K.E., Davis T.H., Doerfler A.M., Bao G., Beeton C., Lagor W.R. (2020). AAV-CRISPR gene editing is negated by pre-existing immunity to Cas9. Mol. Ther..

[22] Charlesworth C.T., Deshpande P.S., Dever D.P., Camarena J., Lemgart V.T., Cromer M.K., Vakulskas C.A., Collingwood M.A., Zhang L., Bode N.M. (2019). Identification of preexisting adaptive immunity to Cas9 proteins in humans. Nat. Med..

[23] Crudele J.M., Chamberlain J.S. (2018). Cas9 immunity creates challenges for CRISPR gene editing therapies. Nat. Commun..

[24] Pickar-Oliver A., Gersbach C.A. (2019). The next generation of CRISPR-Cas technologies and applications. Nat. Rev. Mol. Cell Biol..

[25] Moon S.B., Kim D.Y., Ko J.H., Kim Y.S. (2019). Recent advances in the CRISPR genome editing tool set. Exp. Mol. Med..

[26] Carroll D. (2017). Genome editing: past, present, and future. Yale J. Biol. Med..

[27] Suzuki K., Izpisua Belmonte J.C. (2018). In vivo genome editing via the HITI method as a tool for gene therapy. J. Hum. Genet..

[28] Chang H.H.Y., Pannunzio N.R., Adachi N., Lieber M.R. (2017). Non-homologous DNA end joining and alternative pathways to double-strand break repair. Nat. Rev. Mol. Cell Biol..

[29] Molla K.A., Yang Y. (2019). CRISPR/Cas-mediated base editing: technical considerations and practical applications. Trends Biotechnol..

[30] Anzalone A.V., Randolph P.B., Davis J.R., Sousa A.A., Koblan L.W., Levy J.M., Chen P.J., Wilson C., Newby G.A., Raguram A., Liu D.R. (2019). Search-and-replace genome editing without double-strand breaks or donor DNA. Nature.

[31] Tsai S.Q., Wyvekens N., Khayter C., Foden J.A., Thapar V., Reyon D., Goodwin M.J., Aryee M.J., Joung J.K. (2014). Dimeric CRISPR RNA-guided FokI nucleases for highly specific genome editing. Nat. Biotechnol..

[32] Kleinstiver B.P., Pattanayak V., Prew M.S., Tsai S.Q., Nguyen N.T., Zheng Z., Joung J.K. (2016). High-fidelity CRISPR-Cas9 nucleases with no detectable genome-wide off-target effects. Nature.

[33] Slaymaker I.M., Gao L., Zetsche B., Scott D.A., Yan W.X., Zhang F. (2016). Rationally engineered Cas9 nucleases with improved specificity. Science.

[34] Casini A., Olivieri M., Petris G., Montagna C., Reginato G., Maule G., Lorenzin F., Prandi D., Romanel A., Demichelis F. (2018). A highly specific SpCas9 variant is identified by in vivo screening in yeast. Nat. Biotechnol..

[35] Chen J.S., Dagdas Y.S., Kleinstiver B.P., Welch M.M., Sousa A.A., Harrington L.B., Sternberg S.H., Joung J.K., Yildiz A., Doudna J.A. (2017). Enhanced proofreading governs CRISPR-Cas9 targeting accuracy. Nature.

[36] Kleinstiver B.P., Prew M.S., Tsai S.Q., Topkar V.V., Nguyen N.T., Zheng Z., Gonzales A.P.W., Li Z., Peterson R.T., Yeh J.R.J. (2015). Engineered CRISPR-Cas9 nucleases with altered PAM specificities. Nature.

[37] Fu Y., Sander J.D., Reyon D., Cascio V.M., Joung J.K. (2014). Improving CRISPR-Cas nuclease specificity using truncated guide RNAs. Nat. Biotechnol..

[38] Kocak D.D., Josephs E.A., Bhandarkar V., Adkar S.S., Kwon J.B., Gersbach C.A. (2019). Increasing the specificity of CRISPR systems with engineered RNA secondary structures. Nat. Biotechnol..

[39] Yin H., Song C.Q., Suresh S., Kwan S.Y., Wu Q., Walsh S., Ding J., Bogorad R.L., Zhu L.J., Wolfe S.A. (2018). Partial DNA-guided Cas9 enables genome editing with reduced off-target activity. Nat. Chem. Biol..

[40] Lee J., Bayarsaikhan D., Bayarsaikhan G., Kim J.S., Schwarzbach E., Lee B. (2020). Recent advances in genome editing of stem cells for drug discovery and therapeutic application. Pharmacol. Ther..

[41] You L., Tong R., Li M., Liu Y., Xue J., Lu Y. (2019). Advancements and obstacles of CRISPR-Cas9 technology in translational research. Mol. Ther. Methods Clin. Dev..

[42] Coller B.S. (2019). Ethics of human genome editing. Annu. Rev. Med..

[43] Lea R.A., Niakan K.K. (2019). Human germline genome editing. Nat. Cell Biol..

[44] Ormond K.E., Mortlock D.P., Scholes D.T., Bombard Y., Brody L.C., Faucett W.A., Garrison N.A., Hercher L., Isasi R., Middleton A. (2017). Human germline genome editing. Am. J. Hum. Genet..

[45] Sukari A., Abdallah N., Nagasaka M. (2019). Unleash the power of the mighty T cells-basis of adoptive cellular therapy. Crit. Rev. Oncol. Hematol..

[46] Yee C., Lizee G., Schueneman A.J. (2015). Endogenous T-cell therapy: clinical experience. Cancer J..

[47] Hanahan D., Weinberg R.A. (2011). Hallmarks of cancer: the next generation. Cell.

[48] Pardoll D.M. (2012). The blockade of immune checkpoints in cancer immunotherapy. Nat. Rev. Cancer.

[49] Hargadon K.M., Johnson C.E., Williams C.J. (2018). Immune checkpoint blockade therapy for cancer: an overview of FDA-approved immune checkpoint inhibitors. Int. Immunopharmacol..

[50] Topalian S.L., Hodi F.S., Brahmer J.R., Gettinger S.N., Smith D.C., McDermott D.F., Powderly J.D., Carvajal R.D., Sosman J.A., Atkins M.B. (2012). Safety, activity, and immune correlates of anti-PD-1 antibody in cancer. N. Engl. J. Med..

[51] Brahmer J.R., Tykodi S.S., Chow L.Q., Hwu W.J., Topalian S.L., Hwu P., Drake C.G., Camacho L.H., Kauh J., Odunsi K. (2012). Safety and activity of anti-PD-L1 antibody in patients with advanced cancer. N. Engl. J. Med..

[52] Hamid O., Robert C., Daud A., Hodi F.S., Hwu W.J., Kefford R., Wolchok J.D., Hersey P., Joseph R.W., Weber J.S. (2013). Safety and tumor responses with lambrolizumab (anti-PD-1) in melanoma. N. Engl. J. Med..

[53] Michot J.M., Bigenwald C., Champiat S., Collins M., Carbonnel F., Postel-Vinay S., Berdelou A., Varga A., Bahleda R., Hollebecque A. (2016). Immune-related adverse events with immune checkpoint blockade: a comprehensive review. Eur. J. Cancer.

[54] Ho W.Y., Nguyen H.N., Wolfl M., Kuball J., Greenberg P.D. (2006). In vitro methods for generating CD8^+^ T-cell clones for immunotherapy from the naïve repertoire. J. Immunol. Methods.

[55] Beane J.D., Lee G., Zheng Z., Mendel M., Abate-Daga D., Bharathan M., Black M., Gandhi N., Yu Z., Chandran S. (2015). Clinical scale zinc finger nuclease-mediated gene editing of PD-1 in tumor infiltrating lymphocytes for the treatment of metastatic melanoma. Mol. Ther..

[56] Su S., Hu B., Shao J., Shen B., Du J., Du Y., Zhou J., Yu L., Zhang L., Chen F. (2016). CRISPR-Cas9 mediated efficient PD-1 disruption on human primary T cells from cancer patients. Sci. Rep..

[57] Marotte L., Simon S., Vignard V., Dupre E., Gantier M., Cruard J., Alberge J.B., Hussong M., Deleine C., Heslan J.M. (2020). Increased antitumor efficacy of PD-1-deficient melanoma-specific human lymphocytes. J. Immunother. Cancer.

[58] Menger L., Sledzinska A., Bergerhoff K., Vargas F.A., Smith J., Poirot L., Pule M., Hererro J., Peggs K.S., Quezada S.A. (2016). TALEN-mediated inactivation of PD-1 in tumor-reactive lymphocytes promotes intratumoral T-cell persistence and rejection of established tumors. Cancer Res..

[59] Zhao Z., Shi L., Zhang W., Han J., Zhang S., Fu Z., Cai J. (2017). CRISPR knock out of programmed cell death protein 1 enhances anti-tumor activity of cytotoxic T lymphocytes. Oncotarget.

[60] Lu S., Yang N., He J., Gong W., Lai Z., Xie L., Tao L., Xu C., Wang H., Zhang G. (2019). Generation of cancer-specific cytotoxic PD-1^−^ T cells using liposome-encapsulated CRISPR/Cas system with dendritic/tumor fusion cells. J. Biomed. Nanotechnol..

[61] Wu, S.; Hangzhou Cancer Hospital, Ltd.; Anhui Kedgene Biotechnology Co., Ltd. (2017). PD-1 knockout engineered T cells for advanced esophageal cancer. https://clinicaltrials.gov/ct2/show/NCT03081715.

[62] Lu, Y.; Sichuan University; Chengdu MedGenCell, Co., Ltd. (2016). PD-1 knockout engineered T cells for metastatic non-small cell lung cancer. https://clinicaltrials.gov/ct2/show/NCT02793856.

[63] Chen, S.; Guangzhou Anjie Biomedical Technology Co., Ltd.; University of Technology, Sydney (2018). Therapeutic vaccine plus PD-1 knockout in prostate cancer treatment. https://clinicaltrials.gov/ct2/show/NCT03525652.

[64] Yang, Y.; The Affiliated Nanjing Drum Tower Hospital of Nanjing University Medical School (2017). PD-1 knockout EBV-CTLs for advanced stage Epstein-Barr virus (EBV) associated malignancies. https://clinicaltrials.gov/ct2/show/NCT03044743.

[65] Lu Y., Xue J., Deng T., Zhou X., Yu K., Deng L., Huang M., Yi X., Liang M., Wang Y. (2020). Safety and feasibility of CRISPR-edited T cells in patients with refractory non-small-cell lung cancer. Nat. Med..

[66] Rothlin C.V., Ghosh S. (2020). Lifting the innate immune barriers to antitumor immunity. J. Immunother. Cancer.

[67] Chiossone L., Dumas P.Y., Vienne M., Vivier E. (2018). Natural killer cells and other innate lymphoid cells in cancer. Nat. Rev. Immunol..

[68] Rautela J., Surgenor E., Huntington N.D. (2018). Efficient genome editing of human natural killer cells by CRISPR RNP. bioRxiv.

[69] Pomeroy E.J., Hunzeker J.T., Kluesner M.G., Lahr W.S., Smeester B.A., Crosby M.R., Lonetree C.L., Yamamoto K., Bendzick L., Miller J.S. (2020). A genetically engineered primary human natural killer cell platform for cancer immunotherapy. Mol. Ther..

[70] Ruella M., Kalos M. (2014). Adoptive immunotherapy for cancer. Immunol. Rev..

[71] Liu X., Zhao Y. (2018). CRISPR/Cas9 genome editing: fueling the revolution in cancer immunotherapy. Curr. Res. Transl. Med..

[72] Kershaw M.H., Westwood J.A., Darcy P.K. (2013). Gene-engineered T cells for cancer therapy. Nat. Rev. Cancer.

[73] Cartellieri M., Bachmann M., Feldmann A., Bippes C., Stamova S., Wehner R., Temme A., Schmitz M. (2010). Chimeric antigen receptor-engineered T cells for immunotherapy of cancer. J. Biomed. Biotechnol..

[74] Ouchi Y., Patil A., Tamura Y., Nishimasu H., Negishi A., Paul S.K., Takemura N., Satoh T., Kimura Y., Kurachi M. (2018). Generation of tumor antigen-specific murine CD8^+^ T cells with enhanced anti-tumor activity via highly efficient CRISPR/Cas9 genome editing. Int. Immunol..

[75] Guo X., Jiang H., Shi B., Zhou M., Zhang H., Shi Z., Du G., Luo H., Wu X., Wang Y. (2018). Disruption of PD-1 enhanced the anti-tumor activity of chimeric antigen receptor T cells against hepatocellular carcinoma. Front. Pharmacol..

[76] Hu B., Zou Y., Zhang L., Tang J., Niedermann G., Firat E., Huang X., Zhu X. (2019). Nucleofection with plasmid DNA for CRISPR/Cas9-mediated inactivation of programmed cell death protein 1 in CD133-specific CAR T cells. Hum. Gene Ther..

[77] Hu W., Zi Z., Jin Y., Li G., Shao K., Cai Q., Ma X., Wei F. (2019). CRISPR/Cas9-mediated PD-1 disruption enhances human mesothelin-targeted CAR T cell effector functions. Cancer Immunol. Immunother..

[78] Rupp L.J., Schumann K., Roybal K.T., Gate R.E., Ye C.J., Lim W.A., Marson A. (2017). CRISPR/Cas9-mediated PD-1 disruption enhances anti-tumor efficacy of human chimeric antigen receptor T cells. Sci. Rep..

[79] Zhu H., You Y., Shen Z., Shi L. (2020). EGFRvIII-CAR-T cells with PD-1 knockout have improved anti-glioma activity. Pathol. Oncol. Res..

[80] Maude S.L., Hucks G.E., Seif A.E., Talekar M.K., Teachey D.T., Baniewicz D., Callahan C., Gonzalez V., Nazimuddin F., Gupta M. (2017). The effect of pembrolizumab in combination with CD19-targeted chimeric antigen receptor (CAR) T cells in relapsed acute lymphoblastic leukemia (ALL). J. Clin. Oncol..

[81] Chong E.A., Melenhorst J.J., Lacey S.F., Ambrose D.E., Gonzalez V., Levine B.L., June C.H., Schuster S.J. (2017). PD-1 blockade modulates chimeric antigen receptor (CAR)-modified T cells: refueling the CAR. Blood.

[82] Shang, X.; Third Military Medical University (2017). CD19 CAR and PD-1 knockout engineered T cells for CD19 positive malignant B-cell derived leukemia and lymphoma. https://clinicaltrials.gov/ct2/show/NCT03298828.

[83] Weidong, H.; Chinese PLA General Hospital (2018). Study of PD-1 gene-knocked out mesothelin-directed CAR-T cells with the conditioning of PC in mesothelin positive multiple solid tumors. https://clinicaltrials.gov/ct2/show/NCT03747965.

[84] Chen, S.; Guangzhou Anjie Biomedical Technology Co., Ltd. (2018). CAR T and PD-1 knockout engineered T cells for esophageal cancer. https://clinicaltrials.gov/ct2/show/NCT03706326.

[85] Chen, S.; Guangzhou Anjie Biomedical Technology Co., Ltd.; University of Technology, Sydney (2018). Anti-MUC1 CAR T cells and PD-1 knockout engineered T cells for NSCLC. https://clinicaltrials.gov/ct2/show/NCT03525782.

[86] Guangxun, G.; Xi’An Yufan Biotechnology Co., Ltd. (2019). CRISPR (HPK1) edited CD19-specific CAR-T cells (XYF19 CAR-T cells) for CD19^+^ leukemia or lymphoma. https://www.clinicaltrials.gov/ct2/show/NCT04037566.

[87] Shui J.W., Boomer J.S., Han J., Xu J., Dement G.A., Zhou G., Tan T.H. (2007). Hematopoietic progenitor kinase 1 negatively regulates T cell receptor signaling and T cell-mediated immune responses. Nat. Immunol..

[88] Alzabin S., Pyarajan S., Yee H., Kiefer F., Suzuki A., Burakoff S., Sawasdikosol S. (2010). Hematopoietic progenitor kinase 1 is a critical component of prostaglandin E2-mediated suppression of the anti-tumor immune response. Cancer Immunol. Immunother..

[89] Liu J., Curtin J., You D., Hillerman S., Li-Wang B., Eraslan R., Xie J., Swanson J., Ho C.P., Oppenheimer S. (2019). Critical role of kinase activity of hematopoietic progenitor kinase 1 in anti-tumor immune surveillance. PLoS ONE.

[90] Hernandez S., Qing J., Thibodeau R.H., Du X., Park S., Lee H.M., Xu M., Oh S., Navarro A., Roose-Girma M. (2018). The kinase activity of hematopoietic progenitor kinase 1 is essential for the regulation of T cell function. Cell Rep..

[91] Zhang W., Shi L., Zhao Z., Du P., Ye X., Li D., Cai Z., Han J., Cai J. (2019). Disruption of CTLA-4 expression on peripheral blood CD8 + T cell enhances anti-tumor efficacy in bladder cancer. Cancer Chemother. Pharmacol..

[92] Shi L., Meng T., Zhao Z., Han J., Zhang W., Gao F., Cai J. (2017). CRISPR knock out CTLA-4 enhances the anti-tumor activity of cytotoxic T lymphocytes. Gene.

[93] Jung I.Y., Kim Y.Y., Yu H.S., Lee M., Kim S., Lee J. (2018). CRISPR/Cas9-mediated knockout of DGK improves antitumor activities of human T cells. Cancer Res..

[94] Zhong X.-P., Hainey E.A., Olenchock B.A., Jordan M.S., Maltzman J.S., Nichols K.E., Shen H., Koretzky G.A. (2003). Enhanced T cell responses due to diacylglycerol kinase ζ deficiency. Nat. Immunol..

[95] Zhang Y., Zhang X., Cheng C., Mu W., Liu X., Li N., Wei X., Liu X., Xia C., Wang H. (2017). CRISPR-Cas9 mediated LAG-3 disruption in CAR-T cells. Front. Med..

[96] Singh N., Shi J., June C.H., Ruella M. (2017). Genome-editing technologies in adoptive T cell immunotherapy for cancer. Curr. Hematol. Malig. Rep..

[97] Torikai H., Cooper L.J. (2016). Translational implications for off-the-shelf immune cells expressing chimeric antigen receptors. mol. ther..

[98] Yang Y., Jacoby E., Fry T.J. (2015). Challenges and opportunities of allogeneic donor-derived CAR T cells. Curr. Opin. Hematol..

[99] Torikai H., Reik A., Liu P.Q., Zhou Y., Zhang L., Maiti S., Huls H., Miller J.C., Kebriaei P., Rabinovich B. (2012). A foundation for universal T-cell based immunotherapy: T cells engineered to express a CD19-specific chimeric-antigen-receptor and eliminate expression of endogenous TCR. Blood.

[100] Poirot L., Philip B., Schiffer-Mannioui C., Le Clerre D., Chion-Sotinel I., Derniame S., Potrel P., Bas C., Lemaire L., Galetto R. (2015). Multiplex genome-edited T-cell manufacturing platform for “off-the-shelf” adoptive T-cell immunotherapies. Cancer Res..

[101] Georgiadis C., Preece R., Nickolay L., Etuk A., Petrova A., Ladon D., Danyi A., Humphryes-Kirilov N., Ajetunmobi A., Kim D. (2018). Long terminal repeat CRISPR-CAR-coupled “universal” T cells mediate potent anti-leukemic effects. Mol. Ther..

[102] Qasim W., Zhan H., Samarasinghe S., Adams S., Amrolia P., Stafford S., Butler K., Rivat C., Wright G., Somana K. (2017). Molecular remission of infant B-ALL after infusion of universal TALEN gene-edited CAR T cells. Sci. Transl. Med..

[103] Servier (Institut de Recherches Internationales Servier); ADIR, a Servier Group company (2016). Study of UCART19 in pediatric patients with relapsed/refractory b acute lymphoblastic leukemia. https://clinicaltrials.gov/ct2/show/NCT02808442.

[104] Servier (Institut de Recherches Internationales Servier); ADIR, a Servier Group company (2016). Dose escalation study of UCART19 in adult patients with relapsed refractory B-cell acute lymphoblastic leukaemia. https://clinicaltrials.gov/ct2/show/NCT02746952.

[105] Allogene Therapeutics (2019). Safety and efficacy of ALLO-501 Anti-CD19 allogeneic CAR T cells in adults with relapsed/refractory large B cell or follicular lymphoma (ALPHA). https://clinicaltrials.gov/ct2/show/NCT03939026.

[106] Allogene Therapeutics (2019). Safety and efficacy of ALLO-715 BCMA allogenic CAR T cells in in adults with relapsed or refractory multiple myeloma (UNIVERSAL). https://clinicaltrials.gov/ct2/show/NCT04093596.

[107] Servier (Institut de Recherches Internationales Servier); ADIR, a Servier Group company (2016). A study to evaluate the long-term safety of patients with advanced lymphoid malignancies who have been previously administered with UCART19/ALLO-501. https://clinicaltrials.gov/ct2/show/NCT02735083.

[108] Ren J., Zhang X., Liu X., Fang C., Jiang S., June C.H., Zhao Y. (2017). A versatile system for rapid multiplex genome-edited CAR T cell generation. Oncotarget.

[109] Ren J., Liu X., Fang C., Jiang S., June C.H., Zhao Y. (2017). Multiplex genome editing to generate universal CAR T cells resistant to PD1 inhibition. Clin. Cancer Res..

[110] Liu X., Zhang Y., Cheng C., Cheng A.W., Zhang X., Li N., Xia C., Wei X., Liu X., Wang H. (2017). CRISPR-Cas9-mediated multiplex gene editing in CAR-T cells. Cell Res..

[111] Choi B.D., Yu X., Castano A.P., Darr H., Henderson D.B., Bouffard A.A., Larson R.C., Scarfò I., Bailey S.R., Gerhard G.M. (2019). CRISPR-Cas9 disruption of PD-1 enhances activity of universal EGFRvIII CAR T cells in a preclinical model of human glioblastoma. J. Immunother. Cancer.

[112] Weidong, H.; Chinese PLA General Hospital (2017). A study evaluating UCART019 in patients with relapsed or refractory CD19^+^ leukemia and lymphoma. https://clinicaltrials.gov/ct2/show/NCT03166878.

[113] Eyquem J., Mansilla-Soto J., Giavridis T., van der Stegen S.J., Hamieh M., Cunanan K.M., Odak A., Gönen M., Sadelain M. (2017). Targeting a CAR to the TRAC locus with CRISPR/Cas9 enhances tumour rejection. Nature.

[114] Roth T.L., Puig-Saus C., Yu R., Shifrut E., Carnevale J., Li P.J., Hiatt J., Saco J., Krystofinski P., Li H. (2018). Reprogramming human T cell function and specificity with non-viral genome targeting. Nature.

[115] CRISPR Therapeutics AG (2019). A safety and efficacy study evaluating CTX110 in subjects with relapsed or refractory B-cell malignancies. https://clinicaltrials.gov/ct2/show/NCT04035434.

[116] CRISPR Therapeutics AG (2020). A safety and efficacy study evaluating CTX120 in subjects with relapsed or refractory multiple myeloma. https://clinicaltrials.gov/ct2/show/NCT04244656.

[117] Xu, K.L., and Zheng, J.N.; Nanjing Bioheng Biotech Co., Ltd. (2019). CTA101 UCAR-T cell injection for treatment of relapsed or refractory CD19^+^ B-cell acute lymphoblastic leukemia. https://clinicaltrials.gov/ct2/show/NCT04154709.

[118] Cellectis S.A. (2019). Phase I study of UCART22 in patients with relapsed or refractory CD22^+^ B-cell acute lymphoblastic leukemia (BALLI-01). https://clinicaltrials.gov/ct2/show/NCT04150497.

[119] The First Affiliated Hospital with Nanjing Medical University; Nanjing Bioheng Biotech Co., Ltd. (2019). CTA101 in the treatment of relapsed or refractory diffuse large B-cell lymphoma. https://clinicaltrials.gov/ct2/show/NCT04026100.

[120] Weidong, H.; Chinese PLA General Hospital (2018). A feasibility and safety study of universal dual specificity CD19 and CD20 or CD22 CAR-T cell immunotherapy for relapsed or refractory leukemia and lymphoma. https://clinicaltrials.gov/ct2/show/NCT03398967.

[121] Cellectis S.A. (2017). Study evaluating safety and efficacy of UCART123 in patients with relapsed/ refractory acute myeloid leukemia. https://clinicaltrials.gov/ct2/show/NCT03190278.

[122] Cellectis S.A. (2019). Study evaluating safety and efficacy of UCART targeting CS1 in patients with relapsed/refractory multiple myeloma (MELANI-01). https://clinicaltrials.gov/ct2/show/NCT04142619.

[123] Zhang, X.; Gracell Biotechnologies (Shanghai) Co., Ltd.; 920th Hospital of Joint Logistics Support Force; The Second Affiliated Hospital of Chongqing Medical University; The Affiliated Hospital of Guizhou Medical University; Central South University; The First Affiliated Hospital of Kunming Medical College; The General Hospital of Western Theater Command; Second Affiliated Hospital of Xi’an Jiaotong University; Nanfang Hospital of Southern Medical University; Fujian Medical University Union Hospital; The First Affiliated Hospital of Anhui Medical University; Tang-Du Hospital (2020). Anti-CD19 U-CAR-T cell therapy for B cell hematologic malignancies. https://clinicaltrials.gov/ct2/show/NCT04264039.

[124] Xiang, X.; Gracell Biotechnologies (Shanghai) Co., Ltd.; 920th Hospital of Joint Logistics Support Force; The Second Affiliated Hospital of Chongqing Medical University; The Affiliated Hospital of Guizhou Medical University; Central South University; The First Affiliated Hospital of Kunming Medical College; The General Hospital of Western Theater Command; Second Affiliated Hospital of Xi’an Jiaotong University; Nanfang Hospital of Southern Medical University; Fujian Medical University Union Hospital; The First Affiliated Hospital of Anhui Medical University; Tang-Du Hospital (2020). Anti-CD7 U-CAR-T cell therapy for T/NK cell hematologic malignancies. https://clinicaltrials.gov/ct2/show/NCT04264078.

[125] Shanghai Bioray Laboratory Inc.; Shanghai Tongji Hospital, Tongji University School of Medicine; Second Xiangya Hospital of Central South University (2018). Efficacy and safety evaluation of BCMA-UCART. https://clinicaltrials.gov/ct2/show/NCT03752541.

[126] Shanghai Bioray Laboratory Inc.; The First Affiliated Hospital of Zhengzhou University; Second Xiangya Hospital of Central South University; Shanghai 10th People’s Hospital (2017). Safety and efficacy evaluation of CD19-UCART. https://clinicaltrials.gov/ct2/show/NCT03229876.

[127] Shanghai Longyao Biotechnology Inc. Ltd. (2019). The clinical study of CD19 UCAR-T cells in patients with B-cell acute lymphoblastic leukemia (B-ALL). https://clinicaltrials.gov/ct2/show/NCT04166838.

[128] Ma H., Padmanabhan Iyer S., Parmar S., Gong Y. (2019). Adoptive cell therapy for acute myeloid leukemia. Leuk. Lymphoma.

[129] Kenderian S.S., Ruella M., Shestova O., Klichinsky M., Aikawa V., Morrissette J.J., Scholler J., Song D., Porter D.L., Carroll M. (2015). CD33-specific chimeric antigen receptor T cells exhibit potent preclinical activity against human acute myeloid leukemia. Leukemia.

[130] Gill S., Tasian S.K., Ruella M., Shestova O., Li Y., Porter D.L., Carroll M., Danet-Desnoyers G., Scholler J., Grupp S.A. (2014). Preclinical targeting of human acute myeloid leukemia and myeloablation using chimeric antigen receptor-modified T cells. Blood.

[131] Kim M.Y., Yu K.R., Kenderian S.S., Ruella M., Chen S., Shin T.H., Aljanahi A.A., Schreeder D., Klichinsky M., Shestova O. (2018). Genetic inactivation of CD33 in hematopoietic stem cells to enable CAR T cell immunotherapy for acute myeloid leukemia. Cell.

[132] Knipping F., Osborn M.J., Petri K., Tolar J., Glimm H., von Kalle C., Schmidt M., Gabriel R. (2017). Genome-wide specificity of highly efficient TALENs and CRISPR/Cas9 for T cell receptor modification. Mol. Ther. Methods Clin. Dev..

[133] University of Pennsylvania (2018). NY-ESO-1-redirected CRISPR (TCRendo and PD1) edited T cells (NYCE T cells). https://clinicaltrials.gov/ct2/show/NCT03399448.

[134] Weidong, H.; Chinese PLA General Hospital (2018). Study of CRISPR-Cas9 mediated PD-1 and TCR gene-knocked out mesothelin-directed CAR-T cells in patients with mesothelin positive multiple solid tumors. https://clinicaltrials.gov/ct2/show/NCT03545815.

[135] Stadtmauer E.A., Fraietta J.A., Davis M.M., Cohen A.D., Weber K.L., Lancaster E., Mangan P.A., Kulikovskaya I., Gupta M., Chen F. (2020). CRISPR-engineered T cells in patients with refractory cancer. Science.

[136] Rapoport A.P., Stadtmauer E.A., Binder-Scholl G.K., Goloubeva O., Vogl D.T., Lacey S.F., Badros A.Z., Garfall A., Weiss B., Finklestein J. (2015). NY-ESO-1-specific TCR-engineered T cells mediate sustained antigen-specific antitumor effects in myeloma. Nat. Med..

[137] Robbins P.F., Kassim S.H., Tran T.L., Crystal J.S., Morgan R.A., Feldman S.A., Yang J.C., Dudley M.E., Wunderlich J.R., Sherry R.M. (2015). A pilot trial using lymphocytes genetically engineered with an NY-ESO-1-reactive T-cell receptor: long-term follow-up and correlates with response. Clin. Cancer Res..

[138] D’Angelo S.P., Melchiori L., Merchant M.S., Bernstein D., Glod J., Kaplan R., Grupp S., Tap W.D., Chagin K., Binder G.K. (2018). Antitumor activity associated with prolonged persistence of adoptively transferred NY-ESO-1 ^c259^T cells in synovial sarcoma. Cancer Discov..

[139] Alcantara M., Tesio M., June C.H., Houot R. (2018). CAR T-cells for T-cell malignancies: challenges in distinguishing between therapeutic, normal, and neoplastic T-cells. Leukemia.

[140] Zhou S., Zhu X., Shen N., Li Q., Wang N., You Y., Zhong Z., Cheng F., Zou P., Zhu X. (2019). T cells expressing CD26-specific chimeric antigen receptors exhibit extensive self-antigen-driven fratricide. Immunopharmacol. Immunotoxicol..

[141] Leisegang M., Wilde S., Spranger S., Milosevic S., Frankenberger B., Uckert W., Schendel D.J. (2010). MHC-restricted fratricide of human lymphocytes expressing survivin-specific transgenic T cell receptors. J. Clin. Invest..

[142] Cooper M.L., Choi J., Staser K., Ritchey J.K., Devenport J.M., Eckardt K., Rettig M.P., Wang B., Eissenberg L.G., Ghobadi A. (2018). An “off-the-shelf” fratricide-resistant CAR-T for the treatment of T cell hematologic malignancies. Leukemia.

[143] Gomes-Silva D., Srinivasan M., Sharma S., Lee C.M., Wagner D.L., Davis T.H., Rouce R.H., Bao G., Brenner M.K., Mamonkin M. (2017). CD7-edited T cells expressing a CD7-specific CAR for the therapy of T-cell malignancies. Blood.

[144] Rouce, R.; The Methodist Hospital System; Center for Cell and Gene Therapy, Baylor College of Medicine (2018). Cell therapy for high risk T-cell malignancies using CD7-specific CAR expressed on autologous T cells. https://clinicaltrials.gov/ct2/show/NCT03690011.

[145] Jemal A., Bray F., Center M.M., Ferlay J., Ward E., Forman D. (2011). Global cancer statistics. CA Cancer J. Clin..

[146] Bruni L., Diaz M., Barrionuevo-Rosas L., Herrero R., Bray F., Bosch F.X., de Sanjosé S., Castellsagué X. (2016). Global estimates of human papillomavirus vaccination coverage by region and income level: a pooled analysis. Lancet Glob. Health.

[147] Crosbie E.J., Einstein M.H., Franceschi S., Kitchener H.C. (2013). Human papillomavirus and cervical cancer. Lancet.

[148] Howlader, N., Noone, A.M., Krapcho, M., Miller, D., Bishop, K., Kosary, C.L., Yu, M., Ruhl, J., Tatalovich, Z., Mariotto, A., Lewis, D.R., Chen, H.S., Feuer, E.J., and Cronin, K.A., eds. (2018). SEER Cancer Statistics Review, 1975–2014 (National Cancer Institute), https://seer.cancer.gov/csr/1975_2014/.

[149] zur Hausen H. (2002). Papillomaviruses and cancer: from basic studies to clinical application. Nat. Rev. Cancer.

[150] Sima N., Wang W., Kong D., Deng D., Xu Q., Zhou J., Xu G., Meng L., Lu Y., Wang S., Ma D. (2008). RNA interference against HPV16 E7 oncogene leads to viral E6 and E7 suppression in cervical cancer cells and apoptosis via upregulation of Rb and p53. Apoptosis.

[151] Wang W., Sima N., Kong D., Luo A., Gao Q., Liao S., Li W., Han L., Wang J., Wang S. (2010). Selective targeting of HPV-16 E6/E7 in cervical cancer cells with a potent oncolytic adenovirus and its enhanced effect with radiotherapy in vitro and vivo. Cancer Lett..

[152] Almeida A.M., Queiroz J.A., Sousa F., Sousa Â. (2019). Cervical cancer and HPV infection: ongoing therapeutic research to counteract the action of E6 and E7 oncoproteins. Drug Discov. Today.

[153] Zheng Z.M., Tang S., Tao M. (2005). Development of resistance to RNAi in mammalian cells. Ann. N Y Acad. Sci..

[154] Ding W., Hu Z., Zhu D., Jiang X., Yu L., Wang X., Zhang C., Wang L., Ji T., Li K. (2014). Zinc finger nucleases targeting the human papillomavirus E7 oncogene induce E7 disruption and a transformed phenotype in HPV16/18-positive cervical cancer cells. Clin. Cancer Res..

[155] Hu Z., Ding W., Zhu D., Yu L., Jiang X., Wang X., Zhang C., Wang L., Ji T., Liu D. (2015). TALEN-mediated targeting of HPV oncogenes ameliorates HPV-related cervical malignancy. J. Clin. Invest..

[156] Shankar S., Prasad D., Sanawar R., Das A.V., Pillai M.R. (2017). TALEN based HPV-E7 editing triggers necrotic cell death in cervical cancer cells. Sci. Rep..

[157] Zhen S., Hua L., Takahashi Y., Narita S., Liu Y.H., Li Y. (2014). In vitro and in vivo growth suppression of human papillomavirus 16-positive cervical cancer cells by CRISPR/Cas9. Biochem. Biophys. Res. Commun..

[158] Hu Z., Yu L., Zhu D., Ding W., Wang X., Zhang C., Wang L., Jiang X., Shen H., He D. (2014). Disruption of HPV16-E7 by CRISPR/Cas system induces apoptosis and growth inhibition in HPV16 positive human cervical cancer cells. BioMed Res. Int..

[159] Kennedy E.M., Kornepati A.V., Goldstein M., Bogerd H.P., Poling B.C., Whisnant A.W., Kastan M.B., Cullen B.R. (2014). Inactivation of the human papillomavirus E6 or E7 gene in cervical carcinoma cells by using a bacterial CRISPR/Cas RNA-guided endonuclease. J. Virol..

[160] Ma, D.; Huazhong University of Science and Technology (2017). Study of molecular-targeted therapy using zinc finger nuclease in cervical precancerous lesions. https://clinicaltrials.gov/ct2/show/NCT02800369.

[161] Ma, D.; Huazhong University of Science and Technology (2017). Study of targeted therapy using transcription activator-like effector nucleases in cervical precancerous lesions. https://clinicaltrials.gov/ct2/show/NCT03226470.

[162] Zheng, H.; First Affiliated Hospital, Sun Yat-Sen University; Jingchu University of Technology (2017). A safety and efficacy study of TALEN and CRISPR/Cas9 in the treatment of HPV-related cervical intraepithelial neoplasia. https://clinicaltrials.gov/ct2/show/NCT03057912.

[163] Zhen S., Lu J.J., Wang L.J., Sun X.M., Zhang J.Q., Li X., Luo W.J., Zhao L. (2016). In vitro and in vivo synergistic therapeutic effect of cisplatin with human papillomavirus16 E6/E7 CRISPR/Cas9 on cervical cancer cell line. Transl. Oncol..

[164] Münger K. (2002). The role of human papillomaviruses in human cancers. Front. Biosci..

[165] Lin S.R., Yang H.C., Kuo Y.T., Liu C.J., Yang T.Y., Sung K.C., Lin Y.Y., Wang H.Y., Wang C.C., Shen Y.C. (2014). The CRISPR/Cas9 system facilitates clearance of the intrahepatic HBV templates in vivo. Mol. Ther. Nucleic Acids.

[166] Seeger C., Sohn J.A. (2014). Targeting hepatitis B virus with CRISPR/Cas9. Mol. Ther. Nucleic Acids.

[167] Kennedy E.M., Cullen B.R. (2015). Bacterial CRISPR/Cas DNA endonucleases: a revolutionary technology that could dramatically impact viral research and treatment. Virology.

[168] Zhen S., Hua L., Liu Y.H., Gao L.C., Fu J., Wan D.Y., Dong L.H., Song H.F., Gao X. (2015). Harnessing the clustered regularly interspaced short palindromic repeat (CRISPR)/CRISPR-associated Cas9 system to disrupt the hepatitis B virus. Gene Ther..

[169] Li H., Sheng C., Wang S., Yang L., Liang Y., Huang Y., Liu H., Li P., Yang C., Yang X. (2017). Removal of integrated hepatitis B virus DNA using CRISPR-Cas9. Front. Cell. Infect. Microbiol..

[170] Scott T., Moyo B., Nicholson S., Maepa M.B., Watashi K., Ely A., Weinberg M.S., Arbuthnot P. (2017). ssAAVs containing cassettes encoding SaCas9 and guides targeting hepatitis B virus inactivate replication of the virus in cultured cells. Sci. Rep..

[171] Liu Y., Zhao M., Gong M., Xu Y., Xie C., Deng H., Li X., Wu H., Wang Z. (2018). Inhibition of hepatitis B virus replication via HBV DNA cleavage by Cas9 from *Staphylococcus aureus*. Antiviral Res..

[172] Jiang C., Mei M., Li B., Zhu X., Zu W., Tian Y., Wang Q., Guo Y., Dong Y., Tan X. (2017). A non-viral CRISPR/Cas9 delivery system for therapeutically targeting HBV DNA and *pcsk9* in vivo. Cell Res..

[173] Wang J., Quake S.R. (2014). RNA-guided endonuclease provides a therapeutic strategy to cure latent herpesviridae infection. Proc. Natl. Acad. Sci. USA.

[174] Yuen K.S., Chan C.P., Wong N.M., Ho C.H., Ho T.H., Lei T., Deng W., Tsao S.W., Chen H., Kok K.H., Jin D.Y. (2015). CRISPR/Cas9-mediated genome editing of Epstein-Barr virus in human cells. J. Gen. Virol..

[175] van Diemen F.R., Kruse E.M., Hooykaas M.J., Bruggeling C.E., Schürch A.C., van Ham P.M., Imhof S.M., Nijhuis M., Wiertz E.J., Lebbink R.J. (2016). CRISPR/Cas9-mediated genome editing of herpesviruses limits productive and latent infections. PLoS Pathog..

[176] Yuen K.S., Wang Z.M., Wong N.M., Zhang Z.Q., Cheng T.F., Lui W.Y., Chan C.P., Jin D.Y. (2018). Suppression of Epstein-Barr virus DNA load in latently infected nasopharyngeal carcinoma cells by CRISPR/Cas9. Virus Res..

[177] de Buhr H., Lebbink R.J. (2018). Harnessing CRISPR to combat human viral infections. Curr. Opin. Immunol..

[178] Mbonye U., Karn J. (2017). The molecular basis for human immunodeficiency virus latency. Annu. Rev. Virol..

[179] Chun T.W., Davey R.T., Engel D., Lane H.C., Fauci A.S. (1999). Re-emergence of HIV after stopping therapy. Nature.

[180] Iacob S.A., Iacob D.G., Jugulete G. (2017). Improving the adherence to antiretroviral therapy, a difficult but essential task for a successful HIV treatment-clinical points of view and practical considerations. Front. Pharmacol..

[181] Allers K., Hütter G., Hofmann J., Loddenkemper C., Rieger K., Thiel E., Schneider T. (2011). Evidence for the cure of HIV infection by CCR5Δ32/Δ32 stem cell transplantation. Blood.

[182] Hütter G., Nowak D., Mossner M., Ganepola S., Müssig A., Allers K., Schneider T., Hofmann J., Kücherer C., Blau O. (2009). Long-term control of HIV by CCR5 delta32/delta32 stem-cell transplantation. N. Engl. J. Med..

[183] Wu L., Gerard N.P., Wyatt R., Choe H., Parolin C., Ruffing N., Borsetti A., Cardoso A.A., Desjardin E., Newman W. (1996). CD4-induced interaction of primary HIV-1 gp120 glycoproteins with the chemokine receptor CCR-5. Nature.

[184] Gupta R.K., Abdul-Jawad S., McCoy L.E., Mok H.P., Peppa D., Salgado M., Martinez-Picado J., Nijhuis M., Wensing A.M.J., Lee H. (2019). HIV-1 remission following CCR5Δ32/Δ32 haematopoietic stem-cell transplantation. Nature.

[185] Ioannidis J.P., Rosenberg P.S., Goedert J.J., Ashton L.J., Benfield T.L., Buchbinder S.P., Coutinho R.A., Eugen-Olsen J., Gallart T., Katzenstein T.L., International Meta-Analysis of HIV Host Genetics (2001). Effects of *CCR5-Δ32*, *CCR2-64I*, and *SDF-1 3′A* alleles on HIV-1 disease progression: an international meta-analysis of individual-patient data. Ann. Intern. Med..

[186] Ioannidis J.P., Contopoulos-Ioannidis D.G., Rosenberg P.S., Goedert J.J., De Rossi A., Espanol T., Frenkel L., Mayaux M.J., Newell M.L., Pahwa S.G., HIV Host Genetics International Meta-Analysis Group (2003). Effects of CCR5-delta32 and CCR2-64I alleles on disease progression of perinatally HIV-1-infected children: an international meta-analysis. AIDS.

[187] Mulherin S.A., O’Brien T.R., Ioannidis J.P., Goedert J.J., Buchbinder S.P., Coutinho R.A., Jamieson B.D., Meyer L., Michael N.L., Pantaleo G., International Meta-Analysis of HIV Host Genetics (2003). Effects of CCR5-delta32 and CCR2-64I alleles on HIV-1 disease progression: the protection varies with duration of infection. AIDS.

[188] Perez E.E., Wang J., Miller J.C., Jouvenot Y., Kim K.A., Liu O., Wang N., Lee G., Bartsevich V.V., Lee Y.L. (2008). Establishment of HIV-1 resistance in CD4^+^ T cells by genome editing using zinc-finger nucleases. Nat. Biotechnol..

[189] University of Pennsylvania; Sangamo Therapeutics (2009). Autologous T-cells genetically modified at the CCR5 gene by zinc finger nucleases SB-728 for HIV (zinc-finger). https://clinicaltrials.gov/ct2/show/NCT00842634.

[190] Sangamo Therapeutics (2010). Phase 1 dose escalation study of autologous t-cells genetically modified at the CCR5 gene by zinc finger nucleases in HIV-infected patients. https://clinicaltrials.gov/ct2/show/NCT01044654.

[191] Sangamo Therapeutics (2014). Repeat doses of SB-728mR-T after cyclophosphamide conditioning in HIV-infected subjects on HAART. https://clinicaltrials.gov/ct2/show/NCT02225665.

[192] University of Pennsylvania; National Institute of Allergy and Infectious Diseases (NIAID) (2015). A phase I study of T-cells genetically modified at the CCR5 gene by zinc finger nucleases SB-728mR in HIV-infected patients. https://clinicaltrials.gov/ct2/show/NCT02388594.

[193] Sangamo Therapeutics (2012). Dose escalation study of cyclophosphamide in HIV-infected subjects on HAART receiving SB-728-T. https://clinicaltrials.gov/ct2/show/NCT01543152.

[194] Smith, C.; Case Western Reserve Univeristy; University of California, San Francisco; University of Cincinnati (2018). CCR5-modified CD4^+^ T cells for HIV infection (TRAILBLAZER). https://clinicaltrials.gov/ct2/show/NCT03666871.

[195] Sangamo Therapeutics (2014). Study of autologous T-cells genetically modified at the CCR5 gene by zinc finger nucleases in HIV-infected subjects. https://clinicaltrials.gov/ct2/show/NCT01252641.

[196] Tebas P., Stein D., Tang W.W., Frank I., Wang S.Q., Lee G., Spratt S.K., Surosky R.T., Giedlin M.A., Nichol G. (2014). Gene editing of CCR5 in autologous CD4 T cells of persons infected with HIV. N. Engl. J. Med..

[197] Sangamo Therapeutics (2019). Long-term follow-up of HIV subjects exposed to SB-728-T or SB-728mR-T. https://clinicaltrials.gov/ct2/show/NCT04201782.

[198] Wang C.X., Cannon P.M. (2016). Clinical applications of genome editing to HIV cure. AIDS Patient Care STDS.

[199] Wang C.X., Cannon P.M. (2016). The clinical applications of genome editing in HIV. Blood.

[200] Li L., Krymskaya L., Wang J., Henley J., Rao A., Cao L.F., Tran C.A., Torres-Coronado M., Gardner A., Gonzalez N. (2013). Genomic editing of the HIV-1 coreceptor CCR5 in adult hematopoietic stem and progenitor cells using zinc finger nucleases. Mol. Ther..

[201] Wang J., Exline C.M., DeClercq J.J., Llewellyn G.N., Hayward S.B., Li P.W., Shivak D.A., Surosky R.T., Gregory P.D., Holmes M.C., Cannon P.M. (2015). Homology-driven genome editing in hematopoietic stem and progenitor cells using ZFN mRNA and AAV6 donors. Nat. Biotechnol..

[202] Holt N., Wang J., Kim K., Friedman G., Wang X., Taupin V., Crooks G.M., Kohn D.B., Gregory P.D., Holmes M.C., Cannon P.M. (2010). Human hematopoietic stem/progenitor cells modified by zinc-finger nucleases targeted to CCR5 control HIV-1 in vivo. Nat. Biotechnol..

[203] Krishnan, A.Y.; City of Hope Medical Center; Sangamo Therapeutics;California Institute for Regenerative Medicine (CIRM) (2015). Safety study of zinc finger nuclease CCR5-modified hematopoietic stem/progenitor cells in HIV-1 infected patients. https://clinicaltrials.gov/ct2/show/NCT02500849.

[204] Hu, C.; Affiliated Hospital to Academy of Military Medical Sciences;Peking University; Capital Medical University (2017). Safety of transplantation of CRISPR CCR5 modified CD34^+^ cells in HIV-infected subjects with hematological malignances. https://clinicaltrials.gov/ct2/show/NCT03164135.

[205] Maldini C.R., Ellis G.I., Riley J.L. (2018). CAR T cells for infection, autoimmunity and allotransplantation. Nat. Rev. Immunol..

[206] Tebas, P; University of Pennsylvania (2018). CD4 CAR^+^ ZFN-modified T cells in HIV therapy. https://clinicaltrials.gov/ct2/show/NCT03617198.

[207] Oh D.Y., Jessen H., Kücherer C., Neumann K., Oh N., Poggensee G., Bartmeyer B., Jessen A., Pruss A., Schumann R.R., Hamouda O. (2008). CCR5Δ32 genotypes in a German HIV-1 seroconverter cohort and report of HIV-1 infection in a CCR5Δ32 homozygous individual. PLoS ONE.

[208] Kordelas L., Verheyen J., Beelen D.W., Horn P.A., Heinold A., Kaiser R., Trenschel R., Schadendorf D., Dittmer U., Esser S., Essen HIV AlloSCT Group (2014). Shift of HIV tropism in stem-cell transplantation with *CCR5* Delta32 mutation. N. Engl. J. Med..

[209] Henrich T.J., Hanhauser E., Hu Z., Stellbrink H.J., Noah C., Martin J.N., Deeks S.G., Kuritzkes D.R., Pereyra F. (2015). Viremic control and viral coreceptor usage in two HIV-1-infected persons homozygous for CCR5 Δ32. AIDS.

[210] Murray A.J., Kwon K.J., Farber D.L., Siliciano R.F. (2016). The latent reservoir for HIV-1: how immunologic memory and clonal expansion contribute to HIV-1 persistence. J. Immunol..

[211] Ebina H., Misawa N., Kanemura Y., Koyanagi Y. (2013). Harnessing the CRISPR/Cas9 system to disrupt latent HIV-1 provirus. Sci. Rep..

[212] Hu W., Kaminski R., Yang F., Zhang Y., Cosentino L., Li F., Luo B., Alvarez-Carbonell D., Garcia-Mesa Y., Karn J. (2014). RNA-directed gene editing specifically eradicates latent and prevents new HIV-1 infection. Proc. Natl. Acad. Sci. USA.

[213] Zhu W., Lei R., Le Duff Y., Li J., Guo F., Wainberg M.A., Liang C. (2015). The CRISPR/Cas9 system inactivates latent HIV-1 proviral DNA. Retrovirology.

[214] Kaminski R., Chen Y., Fischer T., Tedaldi E., Napoli A., Zhang Y., Karn J., Hu W., Khalili K. (2016). Elimination of HIV-1 genomes from human T-lymphoid cells by CRISPR/Cas9 gene editing. Sci. Rep..

[215] Wang G., Zhao N., Berkhout B., Das A.T. (2016). A combinatorial CRISPR-Cas9 attack on HIV-1 DNA extinguishes all infectious provirus in infected T cell cultures. Cell Rep..

[216] Liao H.K., Gu Y., Diaz A., Marlett J., Takahashi Y., Li M., Suzuki K., Xu R., Hishida T., Chang C.J. (2015). Use of the CRISPR/Cas9 system as an intracellular defense against HIV-1 infection in human cells. Nat. Commun..

[217] Yin C., Zhang T., Li F., Yang F., Putatunda R., Young W.B., Khalili K., Hu W., Zhang Y. (2016). Functional screening of guide RNAs targeting the regulatory and structural HIV-1 viral genome for a cure of AIDS. AIDS.

[218] Lebbink R.J., de Jong D.C., Wolters F., Kruse E.M., van Ham P.M., Wiertz E.J., Nijhuis M. (2017). A combinational CRISPR/Cas9 gene-editing approach can halt HIV replication and prevent viral escape. Sci. Rep..

[219] Yin L., Hu S., Mei S., Sun H., Xu F., Li J., Zhu W., Liu X., Zhao F., Zhang D. (2018). CRISPR/Cas9 inhibits multiple steps of HIV-1 unfection. Hum. Gene Ther..

[220] Xiao Q., Guo D., Chen S. (2019). Application of CRISPR/Cas9-based gene editing in HIV-1/AIDS therapy. Front. Cell. Infect. Microbiol..

[221] Tsukamoto T. (2019). Gene therapy approaches to functional cure and protection of hematopoietic potential in HIV infection. Pharmaceutics.

[222] Bobbin M.L., Burnett J.C., Rossi J.J. (2015). RNA interference approaches for treatment of HIV-1 infection. Genome Med..

[223] Hartweger H., McGuire A.T., Horning M., Taylor J.J., Dosenovic P., Yost D., Gazumyan A., Seaman M.S., Stamatatos L., Jankovic M., Nussenzweig M.C. (2019). HIV-specific humoral immune responses by CRISPR/Cas9-edited B cells. J. Exp. Med..

[224] Voss J.E., Gonzalez-Martin A., Andrabi R., Fuller R.P., Murrell B., McCoy L.E., Porter K., Huang D., Li W., Sok D. (2019). Reprogramming the antigen specificity of B cells using genome-editing technologies. eLife.

[225] Moffett H.F., Harms C.K., Fitzpatrick K.S., Tooley M.R., Boonyaratanakornkit J., Taylor J.J. (2019). B cells engineered to express pathogen-specific antibodies protect against infection. Sci. Immunol..

[226] Weatherall D.J. (2001). Phenotype-genotype relationships in monogenic disease: lessons from the thalassaemias. Nat. Rev. Genet..

[227] Bank A. (2006). Regulation of human fetal hemoglobin: new players, new complexities. Blood.

[228] Cappellini M.D., Porter J.B., Viprakasit V., Taher A.T. (2018). A paradigm shift on beta-thalassaemia treatment: how will we manage this old disease with new therapies?. Blood Rev..

[229] Shah F.T., Sayani F., Trompeter S., Drasar E., Piga A. (2019). Challenges of blood transfusions in β-thalassemia. Blood Rev..

[230] Bonifazi F., Conte R., Baiardi P., Bonifazi D., Felisi M., Giordano P., Giannuzzi V., Iacono A., Padula R., Pepe A., HTA-THAL Multiregional Registry (2017). Pattern of complications and burden of disease in patients affected by beta thalassemia major. Curr. Med. Res. Opin..

[231] Telfer P. (2009). Update on survival in thalassemia major. Hemoglobin.

[232] Locatelli F., Merli P., Strocchio L. (2016). Transplantation for thalassemia major: alternative donors. Curr. Opin. Hematol..

[233] Tiercy J.M., Claas F. (2013). Impact of HLA diversity on donor selection in organ and stem cell transplantation. Hum. Hered..

[234] Strocchio L., Romano M., Cefalo M.G., Vinti L., Gaspari S., Locatelli F. (2015). Cord blood transplantation in children with hemoglobinopathies. Expert Opin. Orphan Drugs.

[235] Baronciani D., Angelucci E., Potschger U., Gaziev J., Yesilipek A., Zecca M., Orofino M.G., Giardini C., Al-Ahmari A., Marktel S. (2016). Hemopoietic stem cell transplantation in thalassemia: a report from the European Society for Blood and Bone Marrow Transplantation Hemoglobinopathy Registry, 2000–2010. Bone Marrow Transplant..

[236] Piel F.B., Steinberg M.H., Rees D.C. (2017). Sickle cell disease. N. Engl. J. Med..

[237] Kato G.J., Piel F.B., Reid C.D., Gaston M.H., Ohene-Frempong K., Krishnamurti L., Smith W.R., Panepinto J.A., Weatherall D.J., Costa F.F., Vichinsky E.P. (2018). Sickle cell disease. Nat. Rev. Dis. Primers.

[238] Leonard A., Tisdale J.F. (2018). Stem cell transplantation in sickle cell disease: therapeutic potential and challenges faced. Expert Rev. Hematol..

[239] Hsieh M.M., Fitzhugh C.D., Weitzel R.P., Link M.E., Coles W.A., Zhao X., Rodgers G.P., Powell J.D., Tisdale J.F. (2014). Nonmyeloablative HLA-matched sibling allogeneic hematopoietic stem cell transplantation for severe sickle cell phenotype. JAMA.

[240] Hsieh M.M., Kang E.M., Fitzhugh C.D., Link M.B., Bolan C.D., Kurlander R., Childs R.W., Rodgers G.P., Powell J.D., Tisdale J.F. (2009). Allogeneic hematopoietic stem-cell transplantation for sickle cell disease. N. Engl. J. Med..

[241] Fitzhugh C.D., Abraham A.A., Tisdale J.F., Hsieh M.M. (2014). Hematopoietic stem cell transplantation for patients with sickle cell disease: progress and future directions. Hematol. Oncol. Clin. North Am..

[242] Goodman M.A., Malik P. (2016). The potential of gene therapy approaches for the treatment of hemoglobinopathies: achievements and challenges. Ther. Adv. Hematol..

[243] Cavazzana-Calvo M., Payen E., Negre O., Wang G., Hehir K., Fusil F., Down J., Denaro M., Brady T., Westerman K. (2010). Transfusion independence and *HMGA2* activation after gene therapy of human β-thalassaemia. Nature.

[244] Ribeil J.A., Hacein-Bey-Abina S., Payen E., Magnani A., Semeraro M., Magrin E., Caccavelli L., Neven B., Bourget P., El Nemer W. (2017). Gene therapy in a patient with sickle cell disease. N. Engl. J. Med..

[245] Thompson A.A., Walters M.C., Kwiatkowski J., Rasko J.E.J., Ribeil J.A., Hongeng S., Magrin E., Schiller G.J., Payen E., Semeraro M. (2018). Gene therapy in patients with transfusion-dependent β-thalassemia. N. Engl. J. Med..

[246] Marktel S., Scaramuzza S., Cicalese M.P., Giglio F., Galimberti S., Lidonnici M.R., Calbi V., Assanelli A., Bernardo M.E., Rossi C. (2019). Intrabone hematopoietic stem cell gene therapy for adult and pediatric patients affected by transfusion-dependent ß-thalassemia. Nat. Med..

[247] Davé U.P., Jenkins N.A., Copeland N.G. (2004). Gene therapy insertional mutagenesis insights. Science.

[248] Hacein-Bey Abina S., Gaspar H.B., Blondeau J., Caccavelli L., Charrier S., Buckland K., Picard C., Six E., Himoudi N., Gilmour K. (2015). Outcomes following gene therapy in patients with severe Wiskott-Aldrich syndrome. JAMA.

[249] De Ravin S.S., Wu X., Moir S., Anaya-O’Brien S., Kwatemaa N., Littel P., Theobald N., Choi U., Su L., Marquesen M. (2016). Lentiviral hematopoietic stem cell gene therapy for X-linked severe combined immunodeficiency. Sci. Transl. Med..

[250] Biffi A., Montini E., Lorioli L., Cesani M., Fumagalli F., Plati T., Baldoli C., Martino S., Calabria A., Canale S. (2013). Lentiviral hematopoietic stem cell gene therapy benefits metachromatic leukodystrophy. Science.

[251] Sessa M., Lorioli L., Fumagalli F., Acquati S., Redaelli D., Baldoli C., Canale S., Lopez I.D., Morena F., Calabria A. (2016). Lentiviral haemopoietic stem-cell gene therapy in early-onset metachromatic leukodystrophy: an ad-hoc analysis of a non-randomised, open-label, phase 1/2 trial. Lancet.

[252] Eichler F., Duncan C., Musolino P.L., Orchard P.J., De Oliveira S., Thrasher A.J., Armant M., Dansereau C., Lund T.C., Miller W.P. (2017). Hematopoietic stem-cell gene therapy for cerebral adrenoleukodystrophy. N. Engl. J. Med..

[253] Ferrua F., Cicalese M.P., Galimberti S., Giannelli S., Dionisio F., Barzaghi F., Migliavacca M., Bernardo M.E., Calbi V., Assanelli A.A. (2019). Lentiviral haemopoietic stem/progenitor cell gene therapy for treatment of Wiskott-Aldrich syndrome: interim results of a non-randomised, open-label, phase 1/2 clinical study. Lancet Haematol..

[254] Mamcarz E., Zhou S., Lockey T., Abdelsamed H., Cross S.J., Kang G., Ma Z., Condori J., Dowdy J., Triplett B. (2019). Lentiviral gene therapy combined with low-dose busulfan in infants with SCID-X1. N. Engl. J. Med..

[255] Cicalese M.P., Ferrua F., Castagnaro L., Rolfe K., De Boever E., Reinhardt R.R., Appleby J., Roncarolo M.G., Aiuti A. (2018). Gene therapy for adenosine deaminase deficiency: a comprehensive evaluation of short- and medium-term safety. Mol. Ther..

[256] Thein S.L., Menzel S., Lathrop M., Garner C. (2009). Control of fetal hemoglobin: new insights emerging from genomics and clinical implications. Hum. Mol. Genet..

[257] Liu N., Hargreaves V.V., Zhu Q., Kurland J.V., Hong J., Kim W., Sher F., Macias-Trevino C., Rogers J.M., Kurita R. (2018). Direct promoter repression by BCL11A controls the fetal to adult hemoglobin switch. Cell.

[258] Chen Z., Luo H.Y., Steinberg M.H., Chui D.H. (2009). BCL11A represses *HBG* transcription in K562 cells. Blood Cells Mol. Dis..

[259] Sankaran V.G., Xu J., Ragoczy T., Ippolito G.C., Walkley C.R., Maika S.D., Fujiwara Y., Ito M., Groudine M., Bender M.A. (2009). Developmental and species-divergent globin switching are driven by BCL11A. Nature.

[260] Chang K.H., Smith S.E., Sullivan T., Chen K., Zhou Q., West J.A., Liu M., Liu Y., Vieira B.F., Sun C. (2017). Long-term engraftment and fetal globin induction upon *BCL11A* gene editing in bone-marrow-derived CD34^+^ hematopoietic stem and progenitor cells. Mol. Ther. Methods Clin. Dev..

[261] Smith E.C., Luc S., Croney D.M., Woodworth M.B., Greig L.C., Fujiwara Y., Nguyen M., Sher F., Macklis J.D., Bauer D.E., Orkin S.H. (2016). Strict in vivo specificity of the *Bcl11a* erythroid enhancer. Blood.

[262] Psatha N., Reik A., Phelps S., Zhou Y., Dalas D., Yannaki E., Levasseur D.N., Urnov F.D., Holmes M.C., Papayannopoulou T. (2018). Disruption of the BCL11A erythroid enhancer reactivates fetal hemoglobin in erythroid cells of patients with β-thalassemia major. Mol. Ther. Methods Clin. Dev..

[263] Chapin, J.; Vertex Pharmaceuticals Incorporated; CRISPR Therapeutics (2018) A safety and efficacy study evaluating CTX001 in subjects with transfusion-dependent β-thalassemia. https://clinicaltrials.gov/ct2/show/NCT03655678.

[264] Schiller, G., Walters, M., Williams, D., and Smith, A; Sangamo Therapeutics; Sanofi. (2018). A study to assess the safety, tolerability, and efficacy of ST-400 for treatment of transfusion-dependent beta-thalassemia (TDT). https://clinicaltrials.gov/ct2/show/NCT03432364.

[265] Chapin, J.; Vertex Pharmaceuticals Incorporated; CRISPR Therapeutics (2018). A safety and efficacy study evaluating CTX001 in subjects with severe sickle cell disease. https://clinicaltrials.gov/ct2/show/NCT03745287.

[266] Sanofi; Bioverativ Therapeutics Inc. (2018). A study to assess the safety, tolerability, and efficacy of BIVV003 for autologous hematopoietic stem cell transplantation in patients with severe sickle cell disease (BIVV003). https://clinicaltrials.gov/ct2/show/NCT03653247.

[267] Vertex Pharmaceuticals Incorporated; CRISPR Therapeutics (2019). A long-term follow-up study in subjects who received CTX001. https://clinicaltrials.gov/ct2/show/NCT04208529.

[268] Traxler E.A., Yao Y., Wang Y.D., Woodard K.J., Kurita R., Nakamura Y., Hughes J.R., Hardison R.C., Blobel G.A., Li C., Weiss M.J. (2016). A genome-editing strategy to treat β-hemoglobinopathies that recapitulates a mutation associated with a benign genetic condition. Nat. Med..

[269] Lux C.T., Pattabhi S., Berger M., Nourigat C., Flowers D.A., Negre O., Humbert O., Yang J.G., Lee C., Jacoby K. (2018). TALEN-mediated gene editing of *HBG* in human hematopoietic stem cells leads to therapeutic fetal hemoglobin induction. Mol. Ther. Methods Clin. Dev..

[270] Ye L., Wang J., Tan Y., Beyer A.I., Xie F., Muench M.O., Kan Y.W. (2016). Genome editing using CRISPR-Cas9 to create the HPFH genotype in HSPCs: an approach for treating sickle cell disease and β-thalassemia. Proc. Natl. Acad. Sci. USA.

[271] Cai L., Bai H., Mahairaki V., Gao Y., He C., Wen Y., Jin Y.C., Wang Y., Pan R.L., Qasba A. (2018). A universal approach to correct various *HBB* gene mutations in human stem cells for gene therapy of beta-thalassemia and sickle cell disease. Stem Cells Transl. Med..

[272] Wattanapanitch M., Damkham N., Potirat P., Trakarnsanga K., Janan M., U-Pratya Y., Kheolamai P., Klincumhom N., Issaragrisil S. (2018). One-step genetic correction of hemoglobin E/beta-thalassemia patient-derived iPSCs by the CRISPR/Cas9 system. Stem Cell Res. Ther..

[273] Martin R.M., Ikeda K., Cromer M.K., Uchida N., Nishimura T., Romano R., Tong A.J., Lemgart V.T., Camarena J., Pavel-Dinu M. (2019). Highly efficient and marker-free genome editing of human pluripotent stem cells by CRISPR-Cas9 RNP and AAV6 donor-mediated homologous recombination. Cell Stem Cell.

[274] Park S.H., Lee C.M., Dever D.P., Davis T.H., Camarena J., Srifa W., Zhang Y., Paikari A., Chang A.K., Porteus M.H. (2019). Highly efficient editing of the β-globin gene in patient-derived hematopoietic stem and progenitor cells to treat sickle cell disease. Nucleic Acids Res..

[275] Xie F., Ye L., Chang J.C., Beyer A.I., Wang J., Muench M.O., Kan Y.W. (2014). Seamless gene correction of β-thalassemia mutations in patient-specific iPSCs using CRISPR/Cas9 and piggyBac. Genome Res..

[276] Song B., Fan Y., He W., Zhu D., Niu X., Wang D., Ou Z., Luo M., Sun X. (2015). Improved hematopoietic differentiation efficiency of gene-corrected beta-thalassemia induced pluripotent stem cells by CRISPR/Cas9 system. Stem Cells Dev..

[277] Xu P., Tong Y., Liu X.Z., Wang T.T., Cheng L., Wang B.Y., Lv X., Huang Y., Liu D.P. (2015). Both TALENs and CRISPR/Cas9 directly target the HBB IVS2-654 (C > T) mutation in β-thalassemia-derived iPSCs. Sci. Rep..

[278] Niu X., He W., Song B., Ou Z., Fan D., Chen Y., Fan Y., Sun X. (2016). Combining single strand oligodeoxynucleotides and CRISPR/Cas9 to correct gene mutations in β-thalassemia-induced pluripotent stem cells. J. Biol. Chem..

[279] Liu Y., Yang Y., Kang X., Lin B., Yu Q., Song B., Gao G., Chen Y., Sun X., Li X. (2017). One-step biallelic and scarless correction of a β-thalassemia mutation in patient-specific iPSCs without Drug Selection. Mol. Ther. Nucleic Acids.

[280] Allife Medical Science and Technology Co., Ltd. (2018). iHSCs with the gene correction of HBB intervent subjests with β-thalassemia mutations. https://clinicaltrials.gov/ct2/show/NCT03728322.

[281] Mettananda S., Fisher C.A., Hay D., Badat M., Quek L., Clark K., Hublitz P., Downes D., Kerry J., Gosden M. (2017). Editing an α-globin enhancer in primary human hematopoietic stem cells as a treatment for β-thalassemia. Nat. Commun..

[282] VandenDriessche T., Chuah M.K. (2017). Hemophilia gene therapy: ready for prime time?. Hum. Gene Ther..

[283] Franchini M., Mannucci P.M. (2012). Past, present and future of hemophilia: a narrative review. Orphanet J. Rare Dis..

[284] Powell J.S., Ragni M.V., White G.C., Lusher J.M., Hillman-Wiseman C., Moon T.E., Cole V., Ramanathan-Girish S., Roehl H., Sajjadi N. (2003). Phase 1 trial of FVIII gene transfer for severe hemophilia A using a retroviral construct administered by peripheral intravenous infusion. Blood.

[285] Kay M.A., Manno C.S., Ragni M.V., Larson P.J., Couto L.B., McClelland A., Glader B., Chew A.J., Tai S.J., Herzog R.W. (2000). Evidence for gene transfer and expression of factor IX in haemophilia B patients treated with an AAV vector. Nat. Genet..

[286] Manno C.S., Pierce G.F., Arruda V.R., Glader B., Ragni M., Rasko J.J., Ozelo M.C., Hoots K., Blatt P., Konkle B. (2006). Successful transduction of liver in hemophilia by AAV-factor IX and limitations imposed by the host immune response. Nat. Med..

[287] Roth D.A., Tawa N.E., O’Brien J.M., Treco D.A., Selden R.F., Factor VIII Transkaryotic Therapy Study Group (2001). Nonviral transfer of the gene encoding coagulation factor VIII in patients with severe hemophilia A. N. Engl. J. Med..

[288] Sharma R., Anguela X.M., Doyon Y., Wechsler T., DeKelver R.C., Sproul S., Paschon D.E., Miller J.C., Davidson R.J., Shivak D. (2015). In vivo genome editing of the albumin locus as a platform for protein replacement therapy. Blood.

[289] Quon, D., Kuriakose, P.; Sangamo Therapeutics (2016). Ascending dose study of genome editing by zinc finger nuclease therapeutic SB-FIX in subjects with severe hemophilia B. https://clinicaltrials.gov/ct2/show/NCT02695160.

[290] Lyu C., Shen J., Wang R., Gu H., Zhang J., Xue F., Liu X., Liu W., Fu R., Zhang L. (2018). Targeted genome engineering in human induced pluripotent stem cells from patients with hemophilia B using the CRISPR-Cas9 system. Stem Cell Res. Ther..

[291] Sivalingam J., Kenanov D., Han H., Nirmal A.J., Ng W.H., Lee S.S., Masilamani J., Phan T.T., Maurer-Stroh S., Kon O.L. (2016). Multidimensional genome-wide analyses show accurate FVIII integration by ZFN in primary human cells. Mol. Ther..

[292] Li H., Haurigot V., Doyon Y., Li T., Wong S.Y., Bhagwat A.S., Malani N., Anguela X.M., Sharma R., Ivanciu L. (2011). In vivo genome editing restores haemostasis in a mouse model of haemophilia. Nature.

[293] Anguela X.M., Sharma R., Doyon Y., Miller J.C., Li H., Haurigot V., Rohde M.E., Wong S.Y., Davidson R.J., Zhou S. (2013). Robust ZFN-mediated genome editing in adult hemophilic mice. Blood.

[294] Bergmann T., Ehrke-Schulz E., Gao J., Schiwon M., Schildgen V., David S., Schildgen O., Ehrhardt A. (2018). Designer nuclease-mediated gene correction via homology-directed repair in an in vitro model of canine hemophilia B. J. Gene Med..

[295] Ohmori T., Nagao Y., Mizukami H., Sakata A., Muramatsu S.I., Ozawa K., Tominaga S.I., Hanazono Y., Nishimura S., Nureki O., Sakata Y. (2017). CRISPR/Cas9-mediated genome editing via postnatal administration of AAV vector cures haemophilia B mice. Sci. Rep..

[296] Guan Y., Ma Y., Li Q., Sun Z., Ma L., Wu L., Wang L., Zeng L., Shao Y., Chen Y. (2016). CRISPR/Cas9-mediated somatic correction of a novel coagulator factor IX gene mutation ameliorates hemophilia in mouse. EMBO Mol. Med..

[297] He Q., Wang H.H., Cheng T., Yuan W.P., Ma Y.P., Jiang Y.P., Ren Z.H. (2017). Genetic correction and hepatic differentiation of hemophilia B-specific human induced pluripotent stem cells. Chin. Med. Sci. J..

[298] Huai C., Jia C., Sun R., Xu P., Min T., Wang Q., Zheng C., Chen H., Lu D. (2017). CRISPR/Cas9-mediated somatic and germline gene correction to restore hemostasis in hemophilia B mice. Hum. Genet..

[299] Park C.Y., Kim D.H., Son J.S., Sung J.J., Lee J., Bae S., Kim J.H., Kim D.W., Kim J.S. (2015). Functional correction of large factor VIII gene chromosomal inversions in hemophilia A patient-derived iPSCs using CRISPR-Cas9. Cell Stem Cell.

[300] Park C.Y., Kim J., Kweon J., Son J.S., Lee J.S., Yoo J.E., Cho S.R., Kim J.H., Kim J.S., Kim D.W. (2014). Targeted inversion and reversion of the blood coagulation factor 8 gene in human iPS cells using TALENs. Proc. Natl. Acad. Sci. USA.

[301] Park C.Y., Sung J.J., Choi S.H., Lee D.R., Park I.H., Kim D.W. (2016). Modeling and correction of structural variations in patient-derived iPSCs using CRISPR/Cas9. Nat. Protoc..

[302] Wagenblast E., Azkanaz M., Smith S.A., Shakib L., McLeod J.L., Krivdova G., Araújo J., Shultz L.D., Gan O.I., Dick J.E., Lechman E.R. (2019). Functional profiling of single CRISPR/Cas9-edited human long-term hematopoietic stem cells. Nat. Commun..

[303] Genovese P., Schiroli G., Escobar G., Tomaso T.D., Firrito C., Calabria A., Moi D., Mazzieri R., Bonini C., Holmes M.C. (2014). Targeted genome editing in human repopulating haematopoietic stem cells. Nature.

[304] De Ravin S.S., Reik A., Liu P.Q., Li L., Wu X., Su L., Raley C., Theobald N., Choi U., Song A.H. (2016). Targeted gene addition in human CD34^+^ hematopoietic cells for correction of X-linked chronic granulomatous disease. Nat. Biotechnol..

[305] Schiroli G., Ferrari S., Conway A., Jacob A., Capo V., Albano L., Plati T., Castiello M.C., Sanvito F., Gennery A.R. (2017). Preclinical modeling highlights the therapeutic potential of hematopoietic stem cell gene editing for correction of SCID-X1. Sci. Transl. Med..

[306] Sawamoto K., Chen H.H., Alméciga-Díaz C.J., Mason R.W., Tomatsu S. (2018). Gene therapy for mucopolysaccharidoses. Mol. Genet. Metab..

[307] Concolino D., Deodato F., Parini R. (2018). Enzyme replacement therapy: efficacy and limitations. Ital. J. Pediatr..

[308] Tomatsu S., Alméciga-Díaz C.J., Montaño A.M., Yabe H., Tanaka A., Dung V.C., Giugliani R., Kubaski F., Mason R.W., Yasuda E. (2015). Therapies for the bone in mucopolysaccharidoses. Mol. Genet. Metab..

[309] Taylor M., Khan S., Stapleton M., Wang J., Chen J., Wynn R., Yabe H., Chinen Y., Boelens J.J., Mason R.W. (2019). Hematopoietic stem cell transplantation for mucopolysaccharidoses: past, present, and future. Biol. Blood Marrow Transplant..

[310] Schuh R.S., Poletto É., Pasqualim G., Tavares A.M.V., Meyer F.S., Gonzalez E.A., Giugliani R., Matte U., Teixeira H.F., Baldo G. (2018). In vivo genome editing of mucopolysaccharidosis I mice using the CRISPR/Cas9 system. J. Control. Release.

[311] Ellinwood N.M., Ausseil J., Desmaris N., Bigou S., Liu S., Jens J.K., Snella E.M., Mohammed E.E., Thomson C.B., Raoul S. (2011). Safe, efficient, and reproducible gene therapy of the brain in the dog models of Sanfilippo and Hurler syndromes. Mol. Ther..

[312] Motas S., Haurigot V., Garcia M., Marcó S., Ribera A., Roca C., Sánchez X., Sánchez V., Molas M., Bertolin J. (2016). CNS-directed gene therapy for the treatment of neurologic and somatic mucopolysaccharidosis type II (Hunter syndrome). JCI Insight.

[313] Fu H., Dirosario J., Killedar S., Zaraspe K., McCarty D.M. (2011). Correction of neurological disease of mucopolysaccharidosis IIIB in adult mice by rAAV9 trans-blood-brain barrier gene delivery. Mol. Ther..

[314] Sorrentino N.C., D’Orsi L., Sambri I., Nusco E., Monaco C., Spampanato C., Polishchuk E., Saccone P., De Leonibus E., Ballabio A., Fraldi A. (2013). A highly secreted sulphamidase engineered to cross the blood-brain barrier corrects brain lesions of mice with mucopolysaccharidoses type IIIA. EMBO Mol. Med..

[315] Tessitore A., Faella A., O’Malley T., Cotugno G., Doria M., Kunieda T., Matarese G., Haskins M., Auricchio A. (2008). Biochemical, pathological, and skeletal improvement of mucopolysaccharidosis VI after gene transfer to liver but not to muscle. Mol. Ther..

[316] Gurda B.L., De Guilhem De Lataillade A., Bell P., Zhu Y., Yu H., Wang P., Bagel J., Vite C.H., Sikora T., Hinderer C. (2016). Evaluation of AAV-mediated gene therapy for central nervous system disease in canine mucopolysaccharidosis VII. Mol. Ther..

[317] Tardieu M., Zérah M., Gougeon M.L., Ausseil J., de Bournonville S., Husson B., Zafeiriou D., Parenti G., Bourget P., Poirier B. (2017). Intracerebral gene therapy in children with mucopolysaccharidosis type IIIB syndrome: an uncontrolled phase 1/2 clinical trial. Lancet Neurol..

[318] Laoharawee K., DeKelver R.C., Podetz-Pedersen K.M., Rohde M., Sproul S., Nguyen H.O., Nguyen T., St Martin S.J., Ou L., Tom S. (2018). Dose-dependent prevention of metabolic and neurologic disease in murine MPS II by ZFN-mediated in vivo genome editing. Mol. Ther..

[319] Harmatz, P., Heldermon, C., Wilcox, W., Whitley, C., Lau, H., and Leslie, N.; Sangamo Therapeutics (2018). Ascending dose study of genome editing by the zinc finger nuclease (ZFN) therapeutic SB-318 in subjects with MPS I. https://clinicaltrials.gov/ct2/show/NCT02702115.

[320] Burton, B., Whitley, C., Lau, H., Muenzer, J., Prada, C., and Ficcioglu, C; Sangamo Therapeutics (2017). Ascending dose study of genome editing by the zinc finger nuclease (ZFN) therapeutic SB-913 in subjects with MPS II. https://clinicaltrials.gov/ct2/show/NCT03041324.

[321] Kumaran N., Moore A.T., Weleber R.G., Michaelides M. (2017). Leber congenital amaurosis/early-onset severe retinal dystrophy: clinical features, molecular genetics and therapeutic interventions. Br. J. Ophthalmol..

[322] May-Simera H., Nagel-Wolfrum K., Wolfrum U. (2017). Cilia—the sensory antennae in the eye. Prog. Retin. Eye Res..

[323] Jacobson S.G., Cideciyan A.V., Ratnakaram R., Heon E., Schwartz S.B., Roman A.J., Peden M.C., Aleman T.S., Boye S.L., Sumaroka A. (2012). Gene therapy for leber congenital amaurosis caused by RPE65 mutations: safety and efficacy in 15 children and adults followed up to 3 years. Arch. Ophthalmol..

[324] Bainbridge J.W., Smith A.J., Barker S.S., Robbie S., Henderson R., Balaggan K., Viswanathan A., Holder G.E., Stockman A., Tyler N. (2008). Effect of gene therapy on visual function in Leber’s congenital amaurosis. N. Engl. J. Med..

[325] Russell S., Bennett J., Wellman J.A., Chung D.C., Yu Z.F., Tillman A., Wittes J., Pappas J., Elci O., McCague S. (2017). Efficacy and safety of voretigene neparvovec (AAV2-hRPE65v2) in patients with *RPE65*-mediated inherited retinal dystrophy: a randomised, controlled, open-label, phase 3 trial. Lancet.

[326] Maguire A.M., Simonelli F., Pierce E.A., Pugh E.N., Mingozzi F., Bennicelli J., Banfi S., Marshall K.A., Testa F., Surace E.M. (2008). Safety and efficacy of gene transfer for Leber’s congenital amaurosis. N. Engl. J. Med..

[327] Sharif W., Sharif Z. (2017). Leber’s congenital amaurosis and the role of gene therapy in congenital retinal disorders. Int. J. Ophthalmol..

[328] Xu C.L., Cho G.Y., Sengillo J.D., Park K.S., Mahajan V.B., Tsang S.H. (2018). Translation of CRISPR genome surgery to the bedside for retinal diseases. Front. Cell Dev. Biol..

[329] Allergan; Editas Medicine, Inc. (2019). Single ascending dose study in participants with LCA10. https://clinicaltrials.gov/ct2/show/NCT03872479.

[330] Coppieters F., Lefever S., Leroy B.P., De Baere E. (2010). *CEP290*, a gene with many faces: mutation overview and presentation of CEP290*base*. Hum. Mutat..

[331] Ruan G.X., Barry E., Yu D., Lukason M., Cheng S.H., Scaria A. (2017). CRISPR/Cas9-mediated genome editing as a therapeutic approach for Leber congenital amaurosis 10. Mol. Ther..

[332] Maeder M.L., Stefanidakis M., Wilson C.J., Baral R., Barrera L.A., Bounoutas G.S., Bumcrot D., Chao H., Ciulla D.M., DaSilva J.A. (2019). Development of a gene-editing approach to restore vision loss in Leber congenital amaurosis type 10. Nat. Med..

[333] Sanjurjo-Soriano C., Kalatzis V. (2018). Guiding lights in genome editing for inherited retinal disorders: implications for gene and cell therapy. Neural Plast..

[334] Xu C.L., Park K.S., Tsang S.H. (2018). CRISPR/Cas9 genome surgery for retinal diseases. Drug Discov. Today. Technol..

[335] Zhang Z.Y., Thrasher A.J., Zhang F. (2019). Gene therapy and genome editing for primary immunodeficiency diseases. Genes Dis..

[336] Booth C., Romano R., Roncarolo M.G., Thrasher A.J. (2019). Gene therapy for primary immunodeficiency. Hum. Mol. Genet..

[337] VanLith C.J., Guthman R.M., Nicolas C.T., Allen K.L., Liu Y., Chilton J.A., Tritz Z.P., Nyberg S.L., Kaiser R.A., Lillegard J.B., Hickey R.D. (2019). *Ex vivo* hepatocyte reprograming promotes homology-directed DNA repair to correct metabolic disease in mice after transplantation. Hepatol. Commun..

[338] Vaidyanathan S., Salahudeen A.A., Sellers Z.M., Bravo D.T., Choi S.S., Batish A., Le W., Baik R., de la O S., Kaushik M.P. (2020). High-efficiency, selection-free gene repair in airway stem cells from cystic fibrosis patients rescues CFTR function in differentiated epithelia. Cell Stem Cell.

[339] Nissanka N., Moraes C.T. (2020). Mitochondrial DNA heteroplasmy in disease and targeted nuclease-based therapeutic approaches. EMBO Rep..

[340] Zekonyte U., Bacman S.R., Moraes C.T. (2020). DNA-editing enzymes as potential treatments for heteroplasmic mtDNA diseases. J. Intern. Med..

[341] Pankowicz F.P., Barzi M., Legras X., Hubert L., Mi T., Tomolonis J.A., Ravishankar M., Sun Q., Yang D., Borowiak M. (2016). Reprogramming metabolic pathways in vivo with CRISPR/Cas9 genome editing to treat hereditary tyrosinaemia. Nat. Commun..

[342] Villiger L., Grisch-Chan H.M., Lindsay H., Ringnalda F., Pogliano C.B., Allegri G., Fingerhut R., Häberle J., Matos J., Robinson M.D. (2018). Treatment of a metabolic liver disease by in vivo genome base editing in adult mice. Nat. Med..

[343] Lu T., Yang B., Wang R., Qin C. (2020). Xenotransplantation: current status in preclinical research. Front. Immunol..

[344] Ferrari S., Jacob A., Beretta S., Unali G., Albano L., Vavassori V. (2020). Efficient gene editing of human long-term hematopoietic stem cells validated by clonal tracking. Nat. Biotechnol..

